# Engineering Magnetic Heterostructures with Synergistic Regulation of Charge‐Transfer and Spin‐Ordering for Enhanced Water Oxidation

**DOI:** 10.1002/advs.202409842

**Published:** 2024-11-26

**Authors:** Chongyan Hao, Yang Wu, Xiaobo Zheng, Yumeng Du, Yameng Fan, Weikong Pang, Anton Tadich, Shujun Zhang, Thomas Frauenheim, Tianyi Ma, Xiaoning Li, Zhenxiang Cheng

**Affiliations:** ^1^ Institute for Superconducting and Electronic Materials University of Wollongong Wollongong 2500 Australia; ^2^ Bremen Center for Computational Materials Science University of Bremen 28359 Bremen Germany; ^3^ Australian Synchrotron Australian Nuclear Science and Technology Organization Clayton VIC 3168 Australia; ^4^ School of Science Constructor University 28759 Bremen Germany; ^5^ Institute for Advanced Study Chengdu University Chengdu 610106 China; ^6^ School of Science RMIT University Melbourne VIC 3000 Australia

**Keywords:** charge‐transfer, magnetic heterostructures, spin‐ordering, water oxidation

## Abstract

The design of heterojunctions offers a crucial solution for energy conversion and storage challenges, but current research predominantly focuses on charge transfer benefits, often neglecting spin attribute regulation despite the increasing recognition of spin‐sensitivity in many chemical reactions. In this study, a novel magnetic heterostructure, CoFe_2_O_4_@CoFeMo_3_O_8_, is designed to simultaneously modulate charge and spin characteristics, and systematically elucidated their synergistic impact on the oxygen evolution reaction (OER). Experimental results and density functional theory calculations confirmed that the magnetic heterostructure exhibits both charge transfer and spin polarization. It is found that the charge‐transfer behavior enhances conductivity and adsorption ability through band structure regulation. Meanwhile, magnetically polarized electrons promote triplet O_2_ generation and accelerate electron transport via spin‐selective pathways. Moreover, the heterostructure's effective response to external alternating magnetic fields further amplifies the spin‐dependent effect and introduces a magnetothermal effect, locally heating the active sites through spin flip, thereby boosting catalytic activity. Consequently, the OER activity of the magnetic heterostructure is improved by 83.8 times at 1.5 V compared to its individual components. This magnetic heterojunction strategy presents a promising avenue for advanced catalysis through synergistic regulating of charge‐transfer and spin‐ordering.

## Graphical abstract

This study presents a novel magnetic heterostructure, CoFe_2_O_4_@CoFeMo_3_O_8_, that significantly improves conductivity, adsorption, spin‐dependent effect, and magnetothermal effect by synergistically regulating both charge transfer and spin polarization, leading to enhanced oxygen evolution reaction activity.

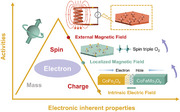

## 1 Introduction

Worldwide, increasing energy demand and pressing environmental issues necessitate efficient and cheap green hydrogen production, with electrochemical water splitting being a pivotal method for a sustainable hydrogen economy.^[^ ^1^, ^2^ ^]^ Yet, the oxygen evolution reaction (OER), one of the two half reactions of water splitting, is a limiting factor for this purpose due to its slow reaction kinetics and high energy consumption.^[^ ^3^, ^4^ ^]^ Traditional noble metal‐based catalysts like IrO_2_ and RuO_2_ are costly and scarce, limiting their widespread use. Transition metal‐based catalysts, particularly transition metal oxides, are emerging as promising alternatives due to their tailorable electronic properties, structural diversity, and natural abundance, making them more viable for large‐scale industrial use.^[^ ^5^, ^6^, ^7^, ^8^, ^9^ ^]^ However, the disadvantages of some oxides, such as poor conductivity and limited active sites, remain limiting factors for their OER efficiency.

To address these challenges, heterogeneous materials composed of two or more types of materials bonded together through physical (primarily van der Waals forces) or chemical bonds is an effective strategy. This approach results in performance advantages greater than those of the individual counterparts due to the presence of unique interfaces and synergistic effects.^[^ ^10^, ^11^, ^12^ ^]^ In addition to combining the superiorities of different materials to neutralize the shortcomings of single component, one of the primary advantages of traditional heterostructure is the regulation of charge by interface. When two different components come into contact, spontaneous band alignment occurs, triggering the redistribution of charges near the interface until the Fermi levels of these materials reach equilibrium. Consequently, electrons and holes gather around the heterojunction and are separated by fully ionized depletion regions, creating an intrinsic potential. This built‐in potential within heterostructure could regulate its electrical conductivity, the electronic structures of active sites, and the adsorption strength of key reaction intermediates.^[^ ^13^, ^14^, ^15^, ^16^ ^]^ These properties have significantly improved the performance of supercapacitors, lithium‐ion batteries (LIBs), water‐splitting, and other energy storage and conversion reactions.^[^ ^17^, ^18^, ^19^, ^20^, ^21^, ^22^, ^23^, ^24^ ^]^

However, given the recognized spin‐sensitive nature of these energy conversion reactions,^[^ ^25^, ^26^ ^]^ there is an urgent need to develop new types of heterostructures capable of regulating spin. Spin is another intrinsic attribute of an electron, and the spin configuration and electron spin interaction, such as the spin‐orbit effect, often determine the electronic structure of the active center, further adjusting the adsorption energy in the reaction steps to achieve optimal conditions (neither too high nor too low).^[^ ^27^, ^28^, ^29^, ^30^, ^31^ ^]^ For example, in oxygen reduction reaction (ORR), the triplet oxygen molecule can interact with the spin polarized electrons in the ferromagnetic catalyst, reducing Coulomb repulsion and thus lowering the reaction barrier for electron transfer between the catalyst and the reactants.^[^ ^32^ ^]^ In the OER, the spin‐polarized electrons could enhance the generation of the triplet oxygen through the quantum spin exchange interaction (QSEI).^[^ ^33^ ^]^ Therefore, constructing tailored new type of heterostructures to simultaneously regulate both electron charge and spin is particularly essential and crucial for the spin‐sensitive associated fields.

Herein, we deliberately designed a CoFe_2_O_4_@CoFeMo_3_O_8_ magnetic heterojunction to explore its possible benefits in the spin‐sensitive OER. The results not only confirm its well‐known effects in the charge transfer, but also reveal that this magnetic heterojunction facilitates the intrinsic spin polarization due to the magnetic proximity effect, which enhances the formation of spin‐selective reaction routes and the efficiency of spin‐dependent electron transport. Notably, it can also effectively respond to external magnetic fields, further improving OER performance through amplifying spin‐dependent effect and beneficial magneto‐thermal effect, which locally heat the active sites through spin flip. Ultimately, the CoFe_2_O_4_@CoFeMo_3_O_8_ magnetic heterostructure exhibits a superior OER performance, with an activity 28.8 times higher than that of pristine CoFeMo_3_O_8_ at a potential of 1.5 V, significantly surpassing most transition metal oxide electrocatalysts.

## 2 Results and Discussion

### 2.1 Structural Characterization of the Heterostructure

**Figure 1:**
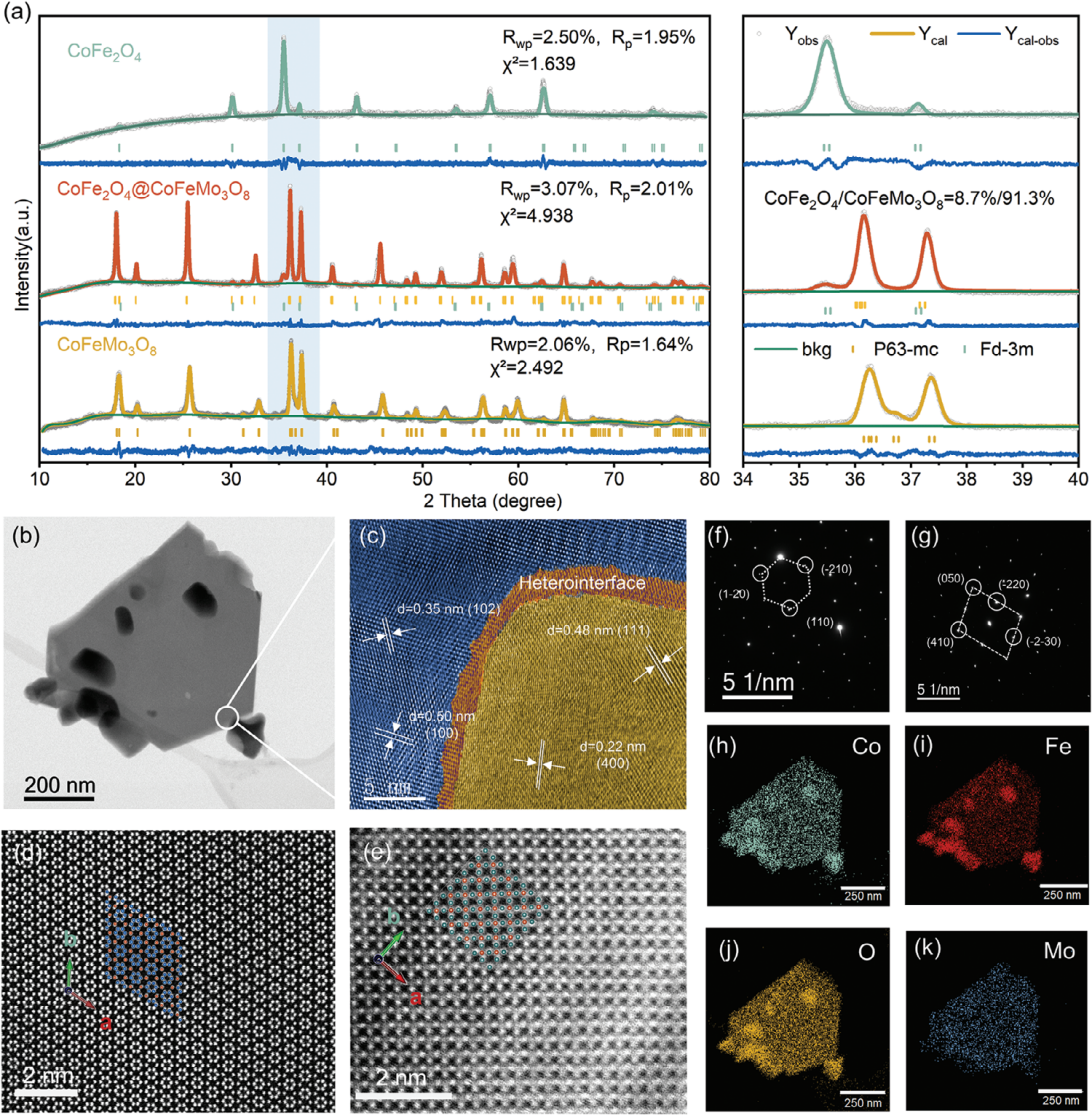
a) XRD patterns and refinements for CoFeMo_3_O_8_, CoFe_2_O_4_@CoFeMo_3_O_8,_ and CoFe_2_O_4_; b) TEM image; c) Colored HRTEM image; HAADF‐STEM image of d) nanosheet region e) nanoparticle region; SAED pattern of f) nanosheet region g) nanoparticle region; h–k) EDS elemental mapping images for Co, Fe, O and Mo for the region shown in (b).

The heterostructure CoFe_2_O_4_@CoFeMo_3_O_8_ and its counterparts, CoFeMo_3_O_8_ and CoFe_2_O_4_, were prepared via a modified hydrothermal‐annealing method, as illustrated in Figure S1 (Supporting Information). The Rietveld‐refined XRD patterns (**Figure** **1**a; Table S1, Supporting Information) indicate that CoFe_2_O_4_ is present as a single spinel phase (space group Fd‐3m). CoFeMo_3_O_8_ is identified in the palmeirite phase (space group *P*6_3_‐mc, as shown in Figure S2, Supporting Information), and CoFe_2_O_4_@CoFeMo_3_O_8_ denotes a combination of both palmeirite and spinel phases. The refinement of the heterostructure reveals that its main phase corresponds to palmeirite (91.3%), with the remaining 8.7% being the secondary spinel phase. The scanning electron microscopy (SEM) images in Figure S3 (Supporting Information) shows the typical morphology of hexagonal nanosheets for CoFeMo_3_O_8_, with an average width ranging from 300 to 600 nm. In the CoFe_2_O_4_@CoFeMo_3_O_8_ heterostructure, some small particles are observed on the surface of the hexagonal CoFeMo_3_O_8_ nanosheets, corresponding to CoFe_2_O_4_. The high‐resolution transmission electron microscope (HRTEM) image and aberration‐corrected high‐angle annual dark‐field scanning transmission electron microscopy (HAADF‐STEM) images in Figure 1b–e further confirm the phase and morphology of CoFe_2_O_4_@CoFeMo_3_O_8_.

The well‐defined heterostructure between CoFeMo_3_O_8_ and CoFe_2_O_4_ is vividly shown in Figure 1c. The CoFeMo_3_O_8_ nanosheet is recorded with the electron beam along the [010] zone axis, in which d spacings of 0.35 and 0.50 nm are well indexed to the (102) and (100) planes, respectively. Correspondingly, the presence of characteristic (111) and (400) lattice planes with 0.48 and 0.22 nm spacing in the nanoparticle agree well with the CoFe_2_O_4_ crystal structure. Figure 1d,e show the good superposition of a typical palmeirite and spinel unit cell projected on the (001) plane (colored dots), respectively. The selected area electron diffraction (SAED) patterns obtained from the corresponding area show sharp diffraction spots in Figure 1f,g, indicating that both CoFeMo_3_O_8_ nanosheets and CoFe_2_O_4_ nanoparticles in the heterostructure exhibit highly single crystallinity. Figure 1h–k is energy dispersive x‐ray spectroscopy (EDS) mapping images, in which Co, Fe, Mo, and O are distributed homogeneously throughout the CoFeMo_3_O_8_ nanosheets. It is also observed that Co, Fe, and O signals are stronger in the nanoparticle regions, indicating that the secondary phase nanoparticles are indeed CoFe_2_O_4_ with enriched Co, Fe and O. Quantitative analysis in Figure S4 (Supporting Information) further confirms the expected ratios of Fe/Co is 1:1 for the nanosheet and 2:1 for the nanoparticle. Corresponding TEM, HAADF‐STEM, SAED, and EDS images of individual CoFeMo_3_O_8_ and CoFe_2_O_4_ are provided in Figures S5 and S6 (Supporting Information).

### 2.2 Electronic Structure and Charge Transfer in the Heterostructure

**Figure 2:**
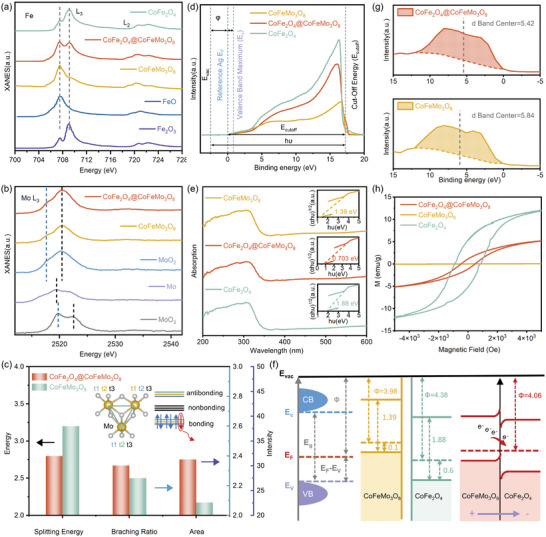
(a) XANES spectra of a) Fe L‐edge; b) Mo L_3_‐edge; c) The comparison of energy splitting, branching intensity ratio and peak area at the Mo L_3_‐edge; d) UPS spectra; e) UV–vis diffuse absorption spectrum, with the transforming plots based on the Kubelka‐Munk function in inset; f) an estimation of the energy band diagram from the UPS and UV–vis spectra; g) valence band spectra; h) M‐H hysteresis loops at room temperature.

As shown in Figure S7 (Supporting Information), the normalized X‐ray absorption near‐edge structure (XANES) spectra of Co L‐edge for all as‐prepared samples are very similar to the reference compound CoO (Co^2+^) rather than Co_2_O_3_ (Co^3+^), suggesting that the Co ions in all of these samples are primarily in the form of Co^2+^. Comparing with FeO and Fe_2_O_3_ (reference for Fe^2+^ and Fe^3+^, respectively), **Figure** **2**a shows that Fe^2+^ is predominant in CoFeMo_3_O_8_, while Fe^3+^ dominates in the CoFe_2_O_4_. As expected, both Fe^2+^ and Fe^3+^ are observed in CoFe_2_O_4_@CoFeMo_3_O_8_. Although the oxidation state of Mo is confirmed as +4 for all catalysts, as shown in Figure 2b, an interesting phenomenon is observed through comparative analysis of the shapes, intensity, and area of the white lines at Mo L_3_‐edges. A significant proportion of Mo d electrons are transferred in the CoFe_2_O_4_@CoFeMo_3_O_8_ heterostructure compared to CoFeMo_3_O_8_, as shown in Figure 2c. More analysis is detailed in Figure S8 and Note S1 (Supporting Information). Related results are further confirmed by X‐ray photoelectron spectroscopy (XPS) spectra of Co 2p, Fe 2p, and Mo 3d as shown in Figures S9 and S10 (Supporting Information). Our conclusion is that the six 4d electrons of Mo occupy all the molecular orbitals of the Mo_3_ trimer cluster in CoFeMo_3_O_8_, resulting in a stable closed shell that is catalytically inert.^[^ ^34^, ^35^ ^]^ However, when incorporated by CoFe_2_O_4_, part of 4d electrons of Mo is transferred due to the formation of a synergistic rectifying interface, resulting in unpaired electrons in Mo. This electron transfer is expected to enhance the hybridization of TM and O, as evidenced by the O spectra, thereby promoting the catalytic activity.^[^ ^36^ ^]^ More details are illustrated in Figure S11 and Note S1 (Supporting Information).

The ultraviolet photoelectron spectroscopy (UPS) and ultraviolet‐visible spectroscopy (UV–vis) provide more information of charge transfer in the heterostructure. Based on the basic principles of UPS (Figures 2d; Figure S12 and Note S2, Supporting Information), the valence band maximum (VBM) position relative to the Fermi level (E_F_) of CoFeMo_3_O_8_, CoFe_2_O_4_@CoFeMo_3_O_8_ and CoFe_2_O_4_ are revealed as 0.10, 0.12 and 0.60 eV, respectively (Figure S13a, Supporting Information). According to the secondary electron cut‐off region represented in the purple shading at the high binding energy range (Figure S13b, Supporting Information), the work function (WF) is estimated to be 3.98, 4.08, and 4.38 eV for CoFeMo_3_O_8_, CoFe_2_O_4_@CoFeMo_3_O_8_ and CoFe_2_O_4_, respectively. The UV–vis spectra, shown in Figure 2e, along with the corresponding transformation by the Kubelka‐Munk function,^[^ ^37^ ^]^ reveal that the bandgap (E_g_) of CoFe_2_O_4_@CoFeMo_3_O_8_ is narrowed to 0.703 eV, which is smaller than that of CoFeMo_3_O_8_ (1.39 eV) and CoFe_2_O_4_ (1.88 eV).

Based on all these experimental results, the band structure of CoFeMo_3_O_8_ and CoFe_2_O_4_ are outlined in Figure 2f. Due to the difference in their WFs, the charge density of each component within the CoFe_2_O_4_@CoFeMo_3_O_8_ is adjusted via interfacial charge exchange.^[^ ^13^ ^]^ Specifically, electrons will automatically flow from CoFeMo_3_O_8_ to CoFe_2_O_4_ to reach equilibrium, creating an internal electric field directed from CoFeMo_3_O_8_ to CoFe_2_O_4_ at the interface. Electrons in CoFeMo_3_O_8_ experience repulsion from this field, leading to an upward band bending. On the contrary, the potential energy of electrons in CoFe_2_O_4_ decreases, causing the bands to bend downward. This refined energy band structure provides superior conductivity, aligning well with the impedance results (will be discussed afterward). Additionally, the partially intrinsic electroneutral components near the boundary become ionized, imparting a positive charge to the nanosheets. These charged sites should be more readily capture OH^−^ during OER, leading to higher performance.^[^ ^13^, ^38^ ^]^

Bader charge analysis quantitatively describes that 0.245 |e| are transferred from CoFeMo_3_O_8_ to CoFe_2_O_4_. This is further verified by the differential charge density (Figure S14, Supporting Information), where the yellow regions indicate electron accumulation and cyan regions indicate electron depletion. A charge‐transfer channel is clearly observed at interface between the two components, which modulates the adsorption and desorption of the intermediates, contributing to a stronger oxidation capacity for the OER. This is corroborated by the d‐band center analysis from XPS valence band spectra (VBS) in Figures 2g and Figure S15 (Supporting Information).

The magnetic property is first measured by magnetic hysteresis loops. As depicted in Figure 2h, both CoFe_2_O_4_@CoFeMo_3_O_8_ and CoFe_2_O_4_ exhibit distinct hysteresis loops with a ferromagnetic (FM) feature at room temperature, while CoFeMo_3_O_8_ displays a linear feature, indicating a paramagnetic (PM) state. The room temperature magnetism observed in CoFe_2_O_4_@CoFeMo_3_O_8_ is primarily contributed by the CoFe_2_O_4_. However, it is noteworthy that despite containing only 8.7% CoFe_2_O_4_ within the heterojunction, the saturation magnetization (Ms) of CoFe_2_O_4_@CoFeMo_3_O_8_ is significantly higher than one‐tenth of that observed for pure CoFe_2_O_4_. This is due to the highly aligned spins on the layered substrate CoFeMo_3_O_8_, induced by the presence of local magnetic domains in the FM CoFe_2_O_4_, a phenomenon commonly known as the magnetic proximity effect.^[^ ^39^ ^]^ The potential benefits of magnetic heterostructure in the spin regulation for OER will be discussed after performance tests.

### 2.3 Catalytic Performance in the Spin‐Sensitive OER

**Figure 3:**
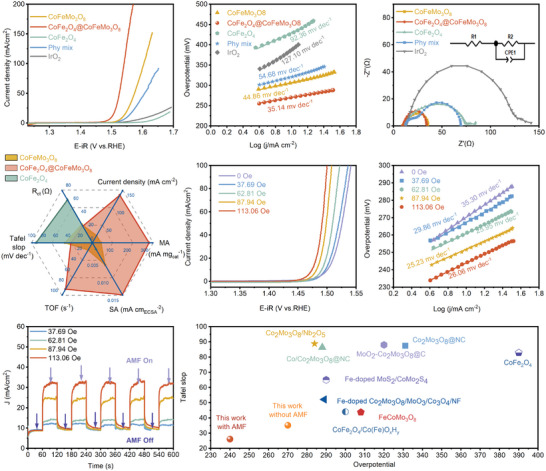
a) LSV curves; b) Tafel plots; c) Nyquist plots measured at 10 mA cm^−2^ Inset is the fitted equivalent circuit; d) Current density, MA, SA, TOF at the potential of 1.55 V versus RHE, Tafel slop, and R_ct_ values; e) LSV curves with different AMF intensities; f) the corresponding Tafel plots with different AMF intensities; g) Magneto‐chronoamperometry response recorded when switched om and then off AMF; h) Overpotentials and Tafel slop of state‐of‐the‐art non‐noble metal‐based OER electrocatalysts.

The OER activities of all samples are evaluated in 1.0 m KOH with 80% iR compensation. The linear sweep voltammetry (LSV) curves, normalized by the electrode area, are shown in **Figure** **3**a. The CoFe_2_O_4_@CoFeMo_3_O_8_ delivers a current density of 10 mA cm^−2^ at an overpotential of 270 mV, which is much lower than those of CoFeMo_3_O_8_ (300 mV), CoFe_2_O_4_ (426 mV), and a reference sample IrO_2_ (387 mV). The performance of a physically mixed sample (8.7%CoFe_2_O_4_ and 91.3%CoFeMo_3_O_8_) is inferior to the heterostructure CoFe_2_O_4_@CoFeMo_3_O_8_. Remarkably, the elaborately in situ grown CoFe_2_O_4_@CoFeMo_3_O_8_ heterostructure requires only an overpotential of 310 mV to achieve a current density of 100 mA cm^−2^. Meanwhile, the lowest Tafel slope of CoFe_2_O_4_@CoFeMo_3_O_8_ (35.14 mV dec^−1^) in Figure 3b indicates its rapid reaction kinetics, with O─O coupling is assumed to be the rate‐determining step (RDS).^[^ ^40^ ^]^ In contrast, the physically mixed sample showed much worse performance, demonstrating the essential role of the heterojunction interface in the performance enhancement.

According to the equivalent circuit diagram in the inset of Figure 3c, the polarization resistance (R_ct_, obtained at current density of 10 mA cm^−2^) is estimated to be ≈25.13, 27.64, 65.35 and 113.9 Ω for CoFe_2_O_4_@CoFeMo_3_O_8_, CoFeMo_3_O_8_, CoFe_2_O_4_ and commercial IrO_2_, respectively (Table S3, Supporting Information). CoFe_2_O_4_@CoFeMo_3_O_8_ exhibits the smallest resistance, indicating a minimal electron transfer barrier when CoFeMo_3_O_8_ is present. As expected, the impedance diagram of physically mixed sample shows two semicircles due to the presence of two independent phases without the well‐defined interaction. To provide a clear comparison between of these catalysts, the current density, mass activity (MA), specific activity (SA), turnover frequency (TOF), Tafel slop, and R_ct_ of these samples are displayed in Figure 3d. Specifically, the MA of CoFe_2_O_4_@CoFeMo_3_O_8_ is ≈7 times higher than that of CoFeMo_3_O_8_, and ≈430 times higher than that of the CoFe_2_O_4_ catalyst at 1.55 V. The SA, normalized by the electrochemically active surface area (ECSA), can exclude the influence of the active surface area.^[^ ^8^, ^41^ ^]^ For CoFe_2_O_4_@CoFeMo_3_O_8_, the SA is ≈2 times and 8 times higher than that of CoFeMo_3_O_8_ and CoFe_2_O_4_, respectively (Figures S16 and S17, Supporting Information). Meanwhile, TOF of CoFe_2_O_4_@CoFeMo_3_O_8_ significantly surpasses that of CoFeMo_3_O_8_ and CoFe_2_O_4_, being ≈6 and 575 times higher, respectively. In conclusion, the as‐prepared CoFe_2_O_4_@CoFeMo_3_O_8_ heterostructure exhibits extremely superior OER activity.

Since the heterostructure exhibits magnetic properties at room temperature, we first analyze its effects on spin regulation by testing LSV curves under varying intensities of alternating magnetic field (AMF). The results in Figure 3e show that the performance of CoFe_2_O_4_@CoFeMo_3_O_8_ is gradually improved as the intensity of the applied AMF increases. Specifically, the overpotential at 10 mA cm^−2^ decreases from 270 to 240 mV when an AMF of 113.06 Oe at 250 kHz is applied (Figure 3f). Consistently, the Tafel slope also decreases to ≈25 mV dec^−1^, which indicates a postponed RDS to the last step.^[^ ^40^ ^]^ This positive effect is more intuitively demonstrated through magneto‐chronoamperometry measurements, which shows remarkably higher OER current density under an AMF (Figure 3g). In contrast, the performances of CoFeMo_3_O_8_ and CoFe_2_O_4_ under AMF show less improvement (Figure S18, Supporting Information). It is noteworthy that the OER activity and Tafel slope of CoFe_2_O_4_@CoFeMo_3_O_8_ has exceeded most recently reported non‐noble metal‐based OER electrocatalysts (Figure 3h; Table S4, Supporting Information), regardless of the presence or absence of AMF.^[^ ^33^, ^42^, ^43^, ^44^, ^45^, ^46^, ^47^, ^48^, ^49^, ^50^, ^51^ ^]^

The incremental performance associated with the AMF can be intuitively attributed to the magnetothermal effect. The continuous realignment of magnetic moments theoretically results in the absorption of energy from the magnetic field, leading to an increase in temperature.^[^ ^52^, ^53^ ^]^ The heated active sites accelerate the adsorption/desorption processes, resulting in faster reaction kinetics and higher OER efficiency. Compared to normal heating, the magnetothermal effect offers several advantages, as it does not significantly raise the temperature of the entire electrolytic cell, effectively reducing the corrosion of the alkaline electrolyte on the entire catalytic system. If this were the only factor, the performance increment should be positively related to the magnetism of the catalysts. However, it's intriguing that CoFe_2_O_4_, which exhibits the strongest magnetism, shows a weaker response to the AMF compared to CoFe_2_O_4_@CoFeMo_3_O_8_, despite the latter having a weaker magnetism. This discrepancy suggests that factors beyond magneto thermal effects may be influencing the materials’ responses.

Interestingly, no obvious decrease in the electrochemical activity is observed during the 10 h CA tests, either with or without AMF (Figure S19, Supporting Information). The HRTEM, XRD, and XPS before and after the OER tests indicate the absence of noticeable surface reconstruction during the OER (Figures S20–S22, Supporting Information). It should be noted that the possibility of the magnetohydrodynamic (MHD) effect in this system is negligible since the AMF has a changing direction. Additionally, the OH⁻ and H₃O⁺ ions do not undergo a physical movement but instead engage in a sequential proton transfer according to the Grotthuss mechanism.^[^ ^33^, ^54^ ^]^ Based on all these experimental evidence and theoretical analysis, it is speculated that the AMF induces an additional effect in the spin regulation, which is discussed in detail below.

### 2.4 Spin Regulation in the Magnetic Heterostructure

**Figure 4:**
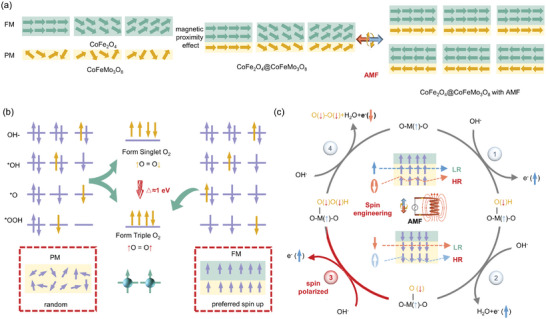
a) Schematic illustration of the generation of the polarized electron between PM CoFeMo_3_O_8_ and FM CoFe_2_O_4_ due to magnetic proximity effect, and further spin electron polarization under an AMF magnetic field; b) schematic diagram of spin alignment in PM and FM catalysts and its acceleration effect on the spin‐electron transfer for the generation of triple oxygen; c) mechanism schematic of CoFe_2_O_4_@CoFeMo_3_O_8_ for OER and the required spin electron transfer in the process of OER under AMF (LR: low resistance; HR: high resistance).

Unlike traditional heterostructure that lack magnetism at room temperature, constructing a magnetic heterostructure induces spin ordering within the catalyst system. The existing magnetic field in magnetic CoFe_2_O_4_ effectively aligns the spins in the paramagnetic CoFeMo_3_O_8_ due to the magnetic proximity effect, thereby enhancing the magnetic properties at the interface (**Figure** **4**a). This alignment act as a spin filter, promoting spin‐selective electron transfer and facilitating triplet oxygen generation during OER, as illustrated in Figure 4b. Specifically, in the CoFeMo_3_O_8_ catalyst without magnetic ordering, electron spins are randomly distributed, allowing equal participation of spin‐up and spin‐down electrons in the OER. This randomness could potentially generate singlet O_2_, which is 1 eV higher in energy than its triplet counterpart, or it may require additional energy for spin flipping to achieve triplet O_2_.^[^ ^20^, ^37^ ^]^ However, in the CoFe_2_O_4_@CoFeMo_3_O_8_ sample with magnetic interface, the electron spins in the CoFeMo_3_O_8_ nanosheets are locally aligned (e.g., spin‐up) and pinned. Only electrons with specific spin direction are allowed to transfer, leading to a preferential extraction of electrons. This spin‐selective process facilitates the turnover of triplet oxygen (↑O = O↑).

This mechanism is deliberately illustrated in Figure 4c. Initially, the singlet OH^−^ bonds with the active sites via chemisorption, and a spin‐up electron (↑) is transferred, forming the ‐O(↓)H. Subsequently, the second electron with the same spin (↑) transports through the catalyst to the external circuit. Since the spin‐polarization process requires less energy, the generated ‐O(↓) further captures OH^−^ to form the triplet intermediate ‐O(↓)O(↓)H species, accompanied by the extraction of another spin‐up electron (↑). Finally, the lone pair electron with spin‐down (↓) is more easily extracted in the last step, forming triplet oxygen during the evolution of O_2_ from ^*^OOH with lower energy.

In the magnetic heterostructure CoFe_2_O_4_@CoFeMo_3_O_8_, while the spin of first three electrons are aligned with the internal magnetic field, the last electron, which should have an anti‐parallel spin, face significant impedance. This is due to enhanced electron scattering, leading to higher resistance and reduced efficiency in electron transport, as depicted in Figure 4c. When an external AMF is applied, the rapid frequency (≈250 kHz) allows it to be considered as a quasi‐constant magnetic field for practical purposes. This could induce instantaneously long‐range FM ordering in the CoFe_2_O_4_@CoFeMo_3_O_8_, thereby amplifying the spin filter effect. This ever‐changing external AMF also facilitates the transfer of electrons with different spin direction during the OER catalysis. It is noteworthy that, this FM‐PM heterostructure helps prevent falloff or agglomeration of catalysts under the local magnetic heating due to the physical rotation within the medium by Brownian relaxation, thereby maintaining catalytic performance.^[^ ^55^ ^]^

## 3 Conclusion

In summary, the innovative magnetic heterostructure strategy introduces both an intrinsic electric field and a local magnetic field by combining magnetic CoFe_2_O_4_ nanoparticles with paramagnetic CoFeMo_3_O_8_ nanosheets. This heterostructure enhances catalyst conductivity and improves adsorption ability of catalytic processes due to the regulation of band structure by the establishment of a traditional electric field. Furthermore, the construction of magnetic heterostructure introduces ferromagnetism through the magnetic proximity effect, adding the spin polarization that accelerates electron transfer and lowers the energy barrier for generating triplet O_2_. Meanwhile, the magnetic heterostructure strongly responds to an external AMF, which amplify the spin‐dependent effect and additionally bring the magnetothermal effects. Ultimately constructing magnetic heterostructures result in outstanding OER performance (240 mV overpotential at current density of 10 mA cm^−2^), making the activity of catalyst improved by 28.8 times at 1.5 V compared to its individual components. This work offers a feasible and efficient strategy for enhancing spin‐sensitive reactions by constructing magnetic heterostructures, thereby advancing the control of heterogeneous electrocatalysts from charge to spin regulation.

## Conflict of Interest

The authors declare no conflict of interest.

## Supporting information



Supporting Information

## Data Availability

The data that support the findings of this study are available from the corresponding author upon reasonable request.
