# Supplementary material for: Engineering Magnetic Heterostructures with Synergistic Regulation of Charge‐Transfer and Spin‐Ordering for Enhanced Water Oxidation
Source: Adv Sci (Weinh). 2024 Nov 26;12(3):2409842. doi: 10.1002/advs.202409842 (PMC11744567; doi:10.1002/advs.202409842)
Supplement: Supplementary file 1 — Supporting Information [file ADVS-12-2409842-s001.docx]

Supporting Information

Engineering Magnetic Heterostructures with Synergistic Regulation of Charge-transfer and Spin-ordering for Enhanced Water Oxidation

Chongyan Hao^a‡^, Yang Wu^b‡^, Xiaobo Zheng^a^, Yumeng Du^a^, Yameng Fan^a^, Weikong Pang^a^, Anton Tadich^c^, Shujun Zhang^a^, Thomas Frauenheim^d,e^, Tianyi Ma^f^, Xiaoning Li^a,f^*, Zhenxiang Cheng^a^*

C. Hao, X. Zheng, Y. Du, Y. Fan, W. Pang, S. Zhang, X. Li, Z. Cheng

Institute for Superconducting and Electronic Materials, University of Wollongong, Wollongong 2500, Australia

E-mail: cheng@uow.edu.au

Y. Wu

Bremen Center for Computational Materials Science, University of Bremen, Bremen 28359, Germany

A. Tadich

Australian Synchrotron, Australian Nuclear Science and Technology Organization, Clayton, Victoria, 3168 Australia

T. Frauenheim

School of Science, Constructor University, Bremen 28759, Germany

Institute for Advanced Study, Chengdu University, Chengdu 610106, China

T. Ma, X. Li

School of Science, RMIT University, Melbourne, VIC 3000, Australia

Email: xiaoning.li@rmit.edu.au

**Experimental section**

**Chemicals**

Cobalt chloride hexahydrate (CoCl_2_⋅6H_2_O, Sigma-Aldrich, purity>98%), Iron(II) sulfate heptahydrate (FeSO_4_⋅7H_2_O, Chem-Supply, Analytical reagent), sodium molybdate dihydrate (Na_2_MoO_4_⋅2H_2_O, Aldrich, purity>99%), 25% ammonia, potassium hydroxide (KOH, Sigma-Aldrich, purity>96.0%) were commercially acquired and employed without further purification.

**Synthesis of** **CoFe_2_O_4_@CoFeMo_3_O_8_**

In a typical synthesis, 1 mmol CoCl_2_⋅6H_2_O, 1 mmol FeSO_4_ · 7H_2_O and 2 mmol Na_2_MoO_4_⋅2H_2_O were dissolved in 17.5 mL deionized water, and then mixed and stirred at 500 rpm for 60 min to form solution. The obtained uniform solution was transferred into a 50 mL Teflon-lined stainless-steel autoclave, and then transferred into an insulated oven at 150 ℃ for 6 h. Followed by furnace cooling to room temperature, the obtained sandy brown product was alternately washed with water and ethanol five times, collected by centrifugation and dried at 60℃ overnight to obtain the precursor. After that, 100 mg of precursor was transferred into a tube furnace, heated to 600 ℃ at a ramp of 5 ℃ min^−1^, and then kept 600℃ for 120 min in a flowing H_2_/Ar (v/v, 5/95) mixture gas. After the cooling to room temperature of furnace, the CoFe_2_O_4_@CoFeMo_3_O_8_ was in-situ synthesized.

**Synthesis of CoFeMo_3_O_8_ and CoFe_2_O_4_**

For the fabrication of reference samples, CoCl_2_⋅6H_2_O, FeSO_4_ · 7H_2_O and Na_2_MoO_4_ were dissolved in 17.5 mL deionized water in different molar ratios of 1:1:3.5 and 1:2:0. During the stirring process, a small amount of ammonia is added to the latter mixture to ensure a consistent PH. Then, the synthesized procedure of precursor is according to the same experimental conditions as CoFe_2_O_4_@CoFeMo_3_O_8_. Finally, these precursors were heated to 600 ℃ for 120 min in a flowing H_2_/Ar (v/v, 5/95) mixture gas and Ar gas, respectively. The resulting black solid products were denoted as CoFeMo_3_O_8_ and CoFe_2_O_4_.

**Catalyst characterization**

The crystal structures of the prepared composites were characterized by a PANalytical Aeris X-ray diffraction apparatus with Cu Kα radiation (λ = 1.5406 Å), followed by Rietveld refinement using GSAS software. The microstructure and micromorphology were observed by scanning transmission electron microscopy (STEM, JEOL JEM-ARM200F) furnished with energy-dispersive X-ray spectroscopy (EDS) mapping and field-emission scanning electron microscopy (SEM, JEOL JSM-7500FA). Raman spectra were obtained using a LabSpec 6 from Horiba Scientific with a 633nm laser, a grating with 300 grooves per mm, and auto calibration. The X-ray absorption near-edge structure (XANES) spectra of the Co L-edge, Fe L-edge, Mo L-edge and O K-edge were collected on the Soft X-ray Spectroscopy Beamline at the Australian Synchrotron (AS, Australia). The X-ray photoelectron spectra (XPS) were recorded on a NEXSA X-ray photoelectron spectrometer (Thermo Fisher Scientific) with monochromatized Al Kα as the excitation source (hν=1486.6 eV) to obtain the surface chemical states, valence band edges and work function of the sample powders. The values of binding energies were calibrated with the C 1s peak of contaminant carbon at 284.80 eV. The Shirly background is subtracted from the measured spectra. The position of the center of the valence band spectra is determined as $\frac{\int N(\varepsilon)\varepsilon d\varepsilon}{\int N(\varepsilon)d\varepsilon}$, where N (ε) is the XPS-intensity after background subtraction. For the ultraviolet photoemission spectra (UPS), a He discharge lamp was used (energy, hν=21.22 eV). Polycrystalline Ag with a work function of 4.26 eV was been used to calibrate the binding energy scale, whilst a negative bias voltage of 10V was added on the sample holder to obtain the secondary electron cutoff measurements and increase the sample signal. The diffuse reflectance spectra (DRS) of sample powders were obtained by ultraviolet-visible (UV-Vis) spectroscopy (Shimadzu UV-3600) with integrated sphere scanning from 300 nm to 800 nm to estimate the energy band gap (E_g_). Magnetic properties were characterized by the vibrating sample magnetometer (VSM) option of a Quantum Design physical property measurement system (PPMS) (Quantum Design, USA).

**Electrochemical testing**

The electrochemical measurements were carried out using an EC-lab electrochemical workstation, with a three-electrode cell configuration. The working electrode used was a 3 mm glassy carbon electrode with an effective electrode area of 0.07065 cm^2^, while platinum foil was used as the counter electrode, and an Hg/HgO electrode served as the reference electrode. The catalyst electrode was prepared through the drop-casting method by dispersing 10 mg of catalyst in 1 ml of ethanol-water (volume ratio = 1:3) solvent, and the addition of 100 μl of 5 wt% Nafion solution as the binder. After ultrasonication for 60 min, 3 μl of the prepared ink was spread onto the surface of the glassy carbon electrode, reaching a loading mass of 38.6 μg_ox_ cm^-2^. In the OER measurements, the electrolyte was purged with high-purity oxygen for approximately 30 minutes before collecting linear sweep voltammograms (LSVs) at a scan rate of 10 mV s^−1^ in a 1.0 M KOH solution. Tafel plots were then obtained from the corresponding polarization curves, and Tafel slopes (b) were calculated using the Tafel equation (η = *b* logj + *a*), where η is the overpotential, *j* is the anodic current density, and *a* and *b* are parameters. Electrochemical impedance spectroscopy (EIS) measurements were recorded for all samples at 10 mA cm^-2^ within the frequency range from 100 kHz to 0.1 Hz. The electrochemically active surface area (ECSA) was estimated by the electrochemical double-layer capacitance (C_dl_), which was measured by cyclic voltammograms (CV) collected between 10 and 60 mV s^-1^ in the non-Faradaic potential region in 1 M KOH. The ECSA was calculated according to the following equation: ECSA = C_dl_/C_s_, where C_s_ represent the specific capacitance (0.040 mF cm^-2^)^[1–3]^. The turnover frequency (TOF) is calculated from the equation below: TOF=j x A /(4 x F x n), Where j is the current density, A is the surface area of the electrode, the factor 4 means that 4 electrons are required to form one oxygen molecular, F is the Faraday constant (96485.3 C mol^-1^ ), and n is the mole number of active sites on the electrode. To convert all polarization curves to the reversible hydrogen electrode (RHE) scale with iR-compensation, the following equation was used: E (vs. RHE) = E (vs. Hg/HgO) + E_0_ (Hg/HgO) + 0.059 × pH - iR, where E (vs. RHE) is the converted potential versus RHE, E_0_ (Hg/HgO) = 0.098V, E (vs. Hg/HgO) is the measured potential against the Hg/HgO reference, and the solution resistance value (R_s_) was resolved from the Nyquist plots. The chronoamperometric (CA) curves at the current density near 10 mA cm-2 were tested on the carbon cloth (1 cm x1.5 cm), using 50 μL of the electrocatalyst ink to prepare the working electrodes (1 cm x1 cm).

**DFT calculation method**

All density functional theory (DFT) computations with the projector augmented wave (PAW) method were conducted by using the Vienna ab initio simulation package (VASP)^[4,5]^. The spin-polarization was considered in all computations. Electron-ion interactions were described using standard PAW potentials, with valence configurations of 4s^1^3d^8^ for Co, 4s^1^3d^7^ for Fe, 4p^6^5s^1^4d^5^ for Mo and 2s^2^2p^2^ for O. A plane-wave basis set was employed to expand the smooth part of wave functions with a cut-off kinetic energy of 520 eV. For the electron-electron exchange and correlation interactions, the functional parameterized by Perdew-Burke-Ernzerhhof (PBE)^[6]^, a form of the general gradient approximation (GGA), was used throughout. Because of the large Coulombic repulsion between the localized d electrons of TMs, the DFT + U method was used to correct the material properties of TM oxides, particularly for magnetic ground states and electronic structures^[7]^. The U-J values of Co, Fe and Mo were set as 3.32, 5.3 and 4.38 from our previous studies. A gamma-centered (6×6×3 for CoFeMo_3_O_8_; 3×3×3 for CoFe_2_O_4_; 3×3×1 for CoFe_2_O_4_@CoFeMo_3_O_8_）k-points mesh k-point mesh was used for the Brillion zone sampling. The energy and force convergence of 10^-5^ eV and 0.01 eV/Å was employed to optimize the electronic and atomic structures, respectively. Bader charge analysis was used to understand the charge transfer between metals^[8]^.

**Supplementary Figures**


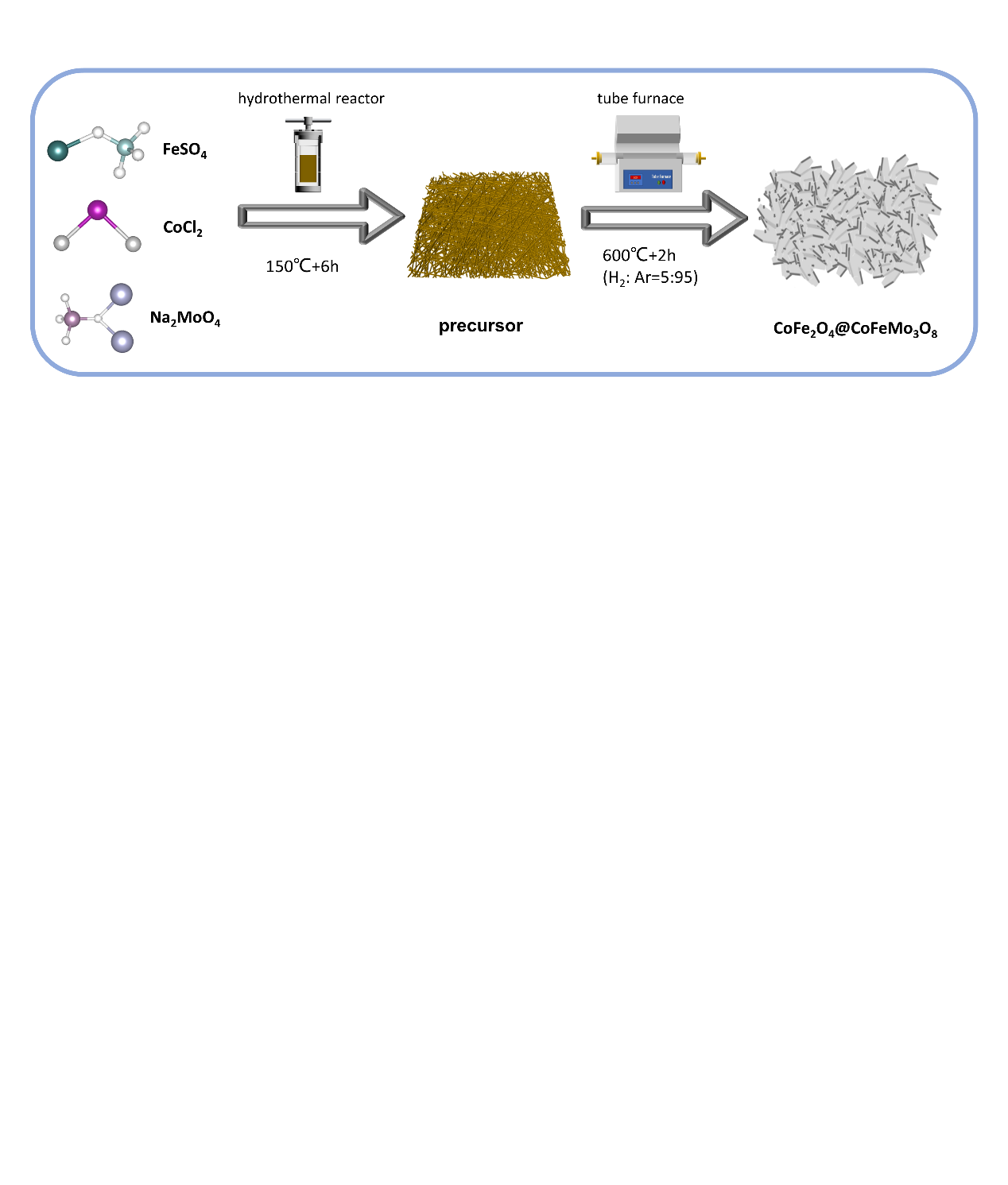


**Figure S1** Schematic illustration of the preparation process of CoFe_2_O_4_@CoFeMo_3_O_8_.

**
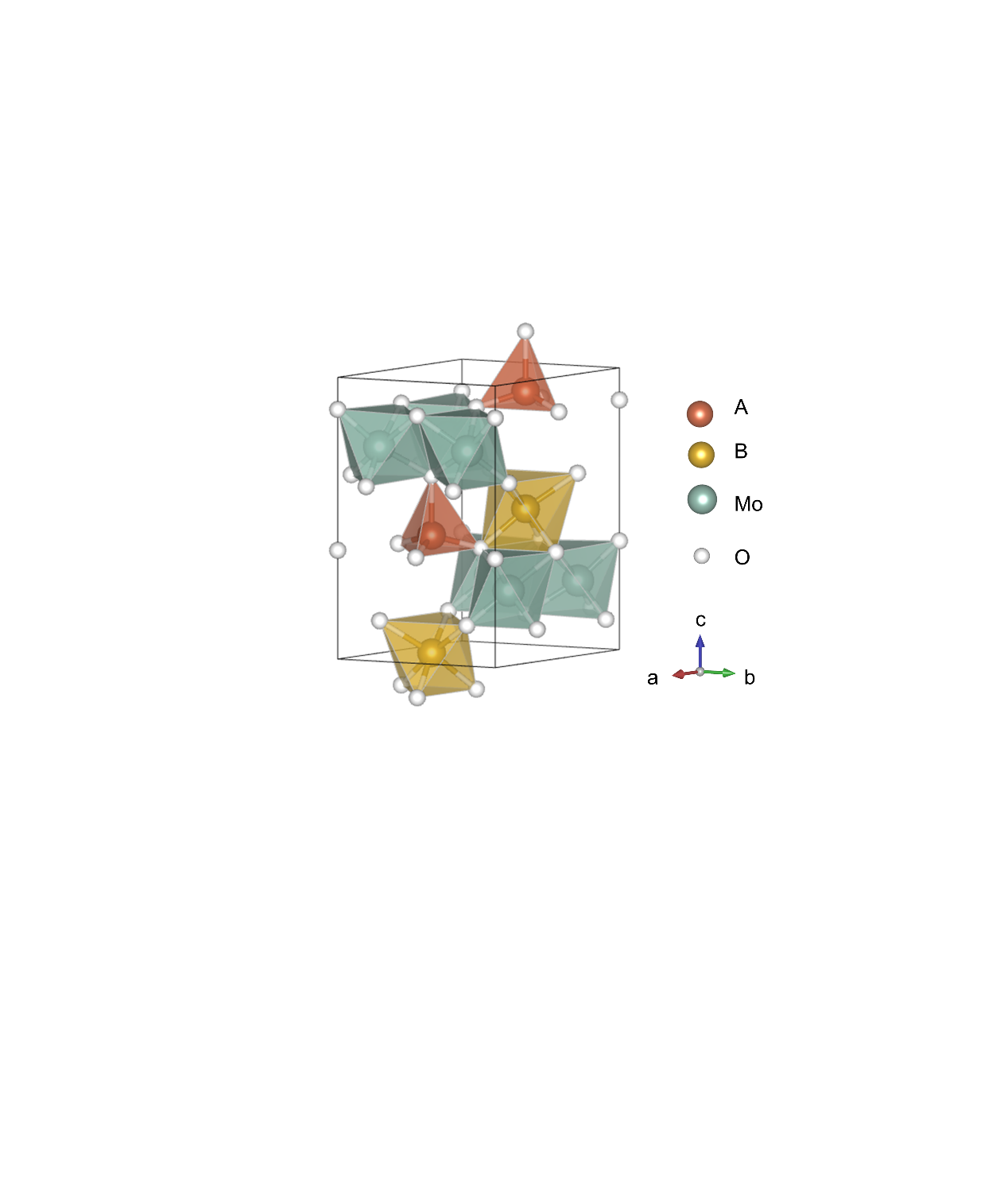
**

**Figure S2** Crystal structure of CoFeMo_3_O_8_.

_
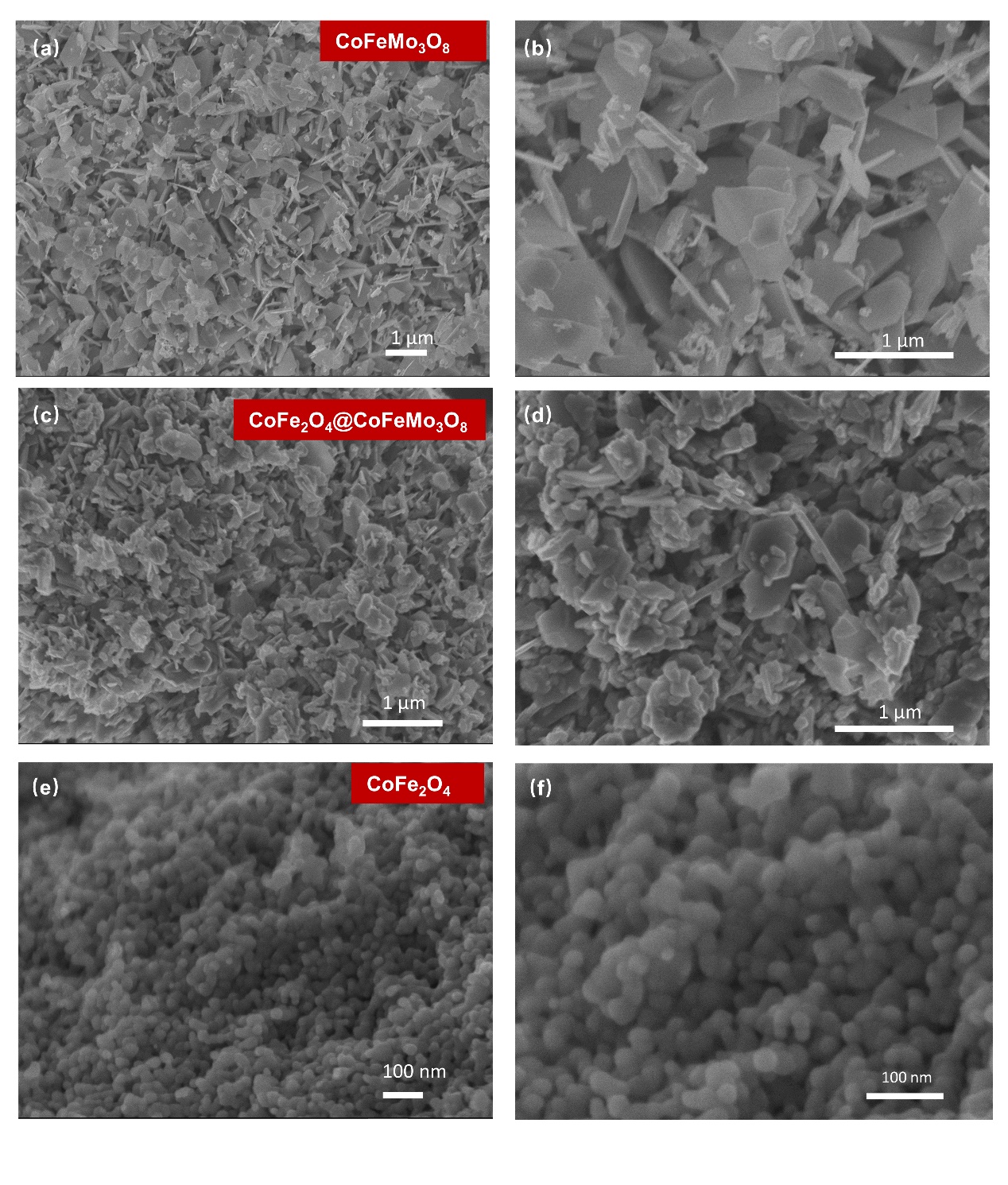
_

**Figure S3** SEM images of as-prepared samples. (a-b) CoFeMo_3_O_8_; (c-d) CoFe_2_O_4_@CoFeMo_3_O_8_; (e-f) CoFe_2_O_4_.


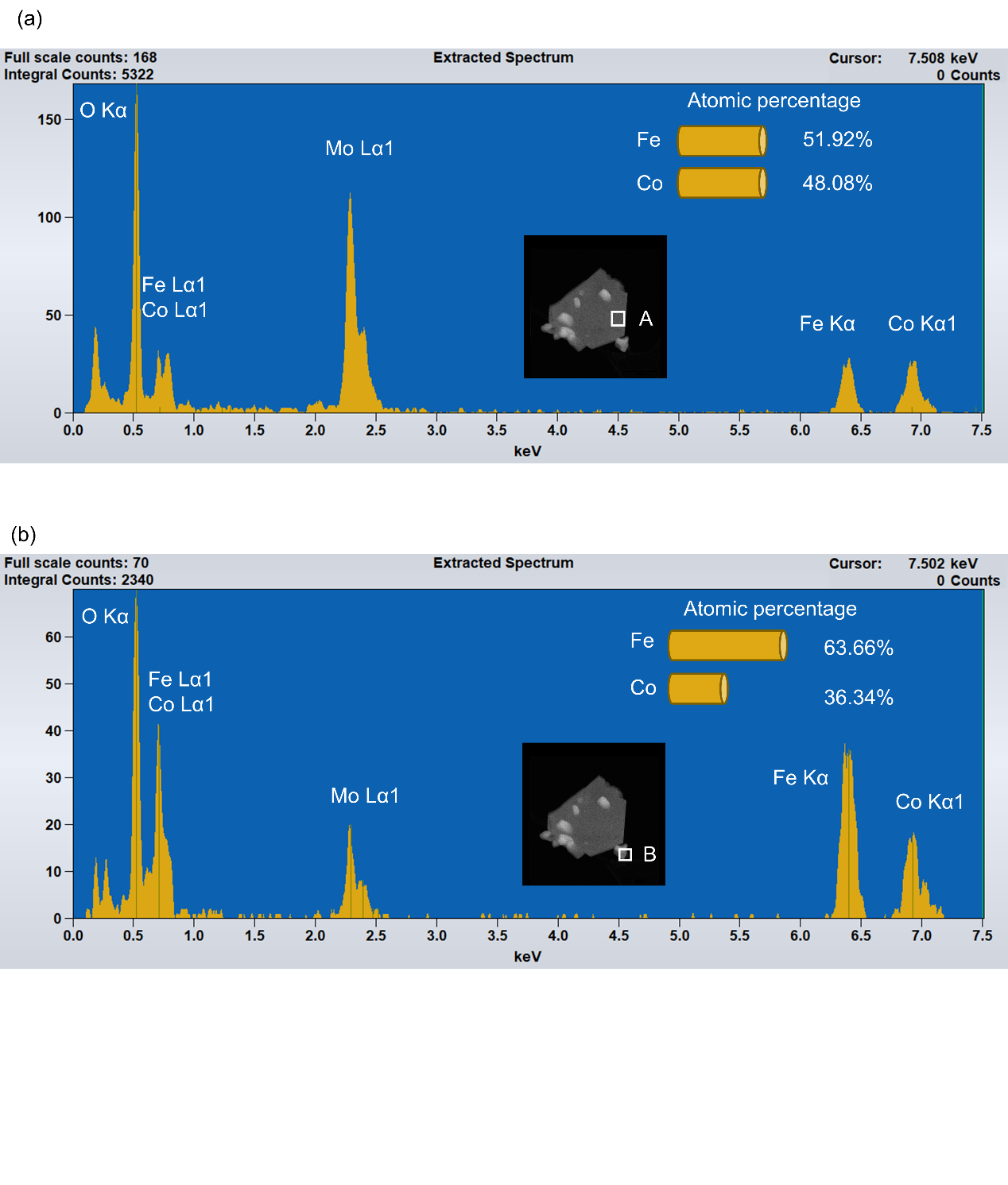


**Figure S4** Area EDS spectrum and the corresponding atomic percentage of Co and Fe elements in (a) the layer region and (b) particle region for CoFe_2_O_4_@CoFeMo_3_O_8_.


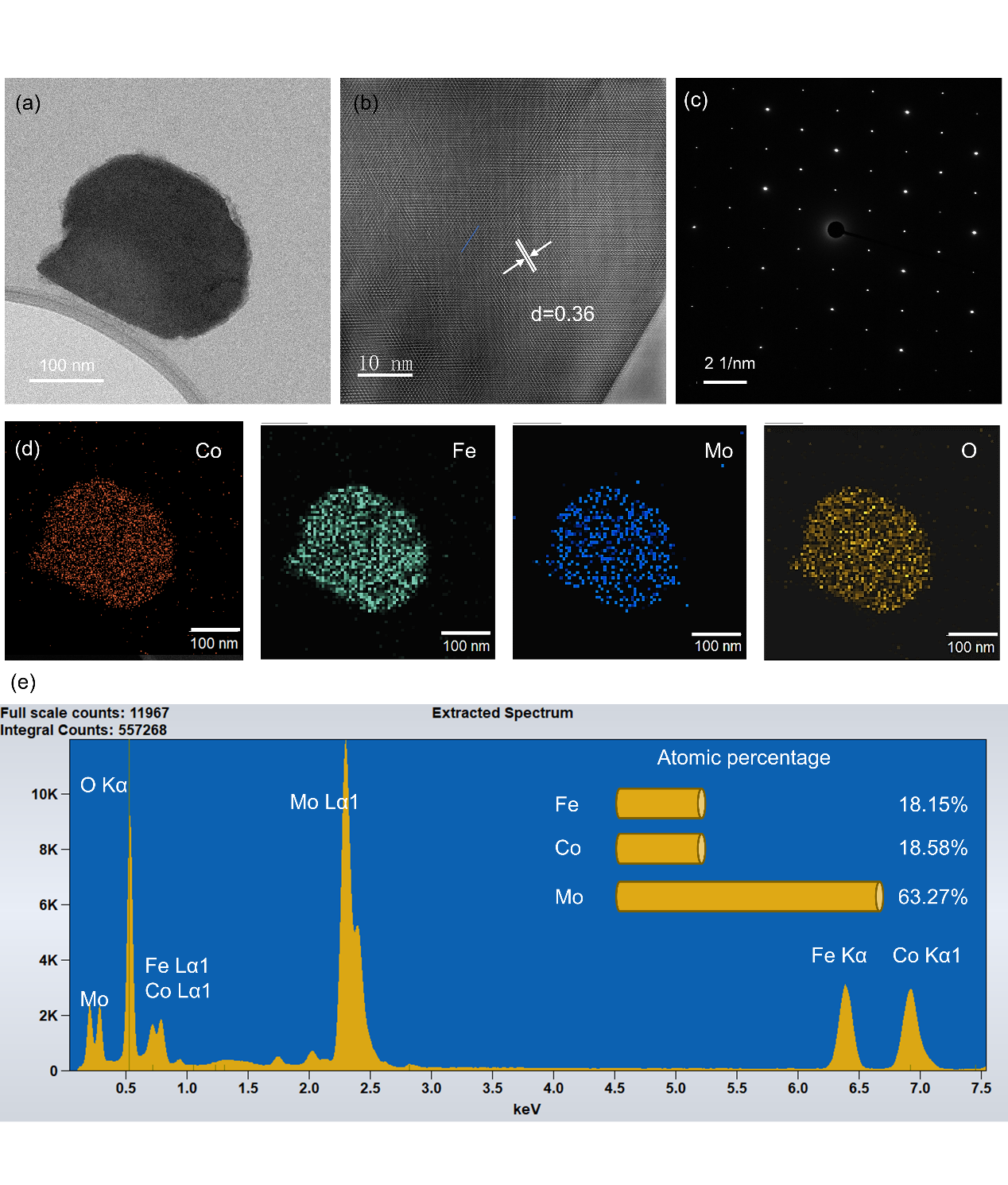


**Figure S5** (a) TEM image; (b) HRTEM image; (c) SAED pattern; (d) EDS element mapping; (e) EDS area spectrum and the corresponding atomic percentage of Co, Fe and Mo elements for CoFeMo_3_O_8_.


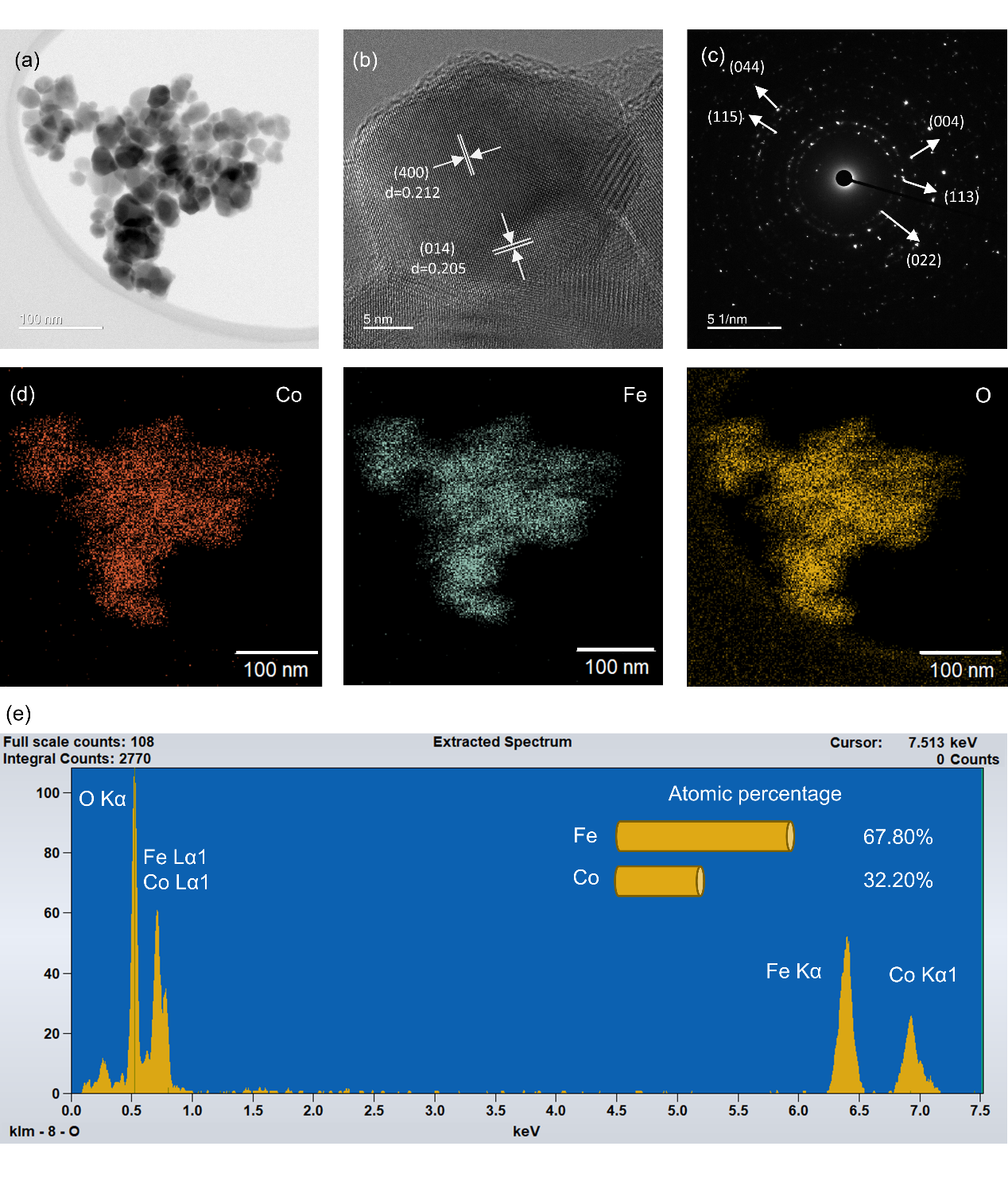


**Figure S6** (a) TEM image; (b) HRTEM image; (c) SAED pattern; (d) EDS element mapping; (e) EDS area spectrum and the corresponding atomic percentage of Co and Fe elements for CoFe_2_O_4_.


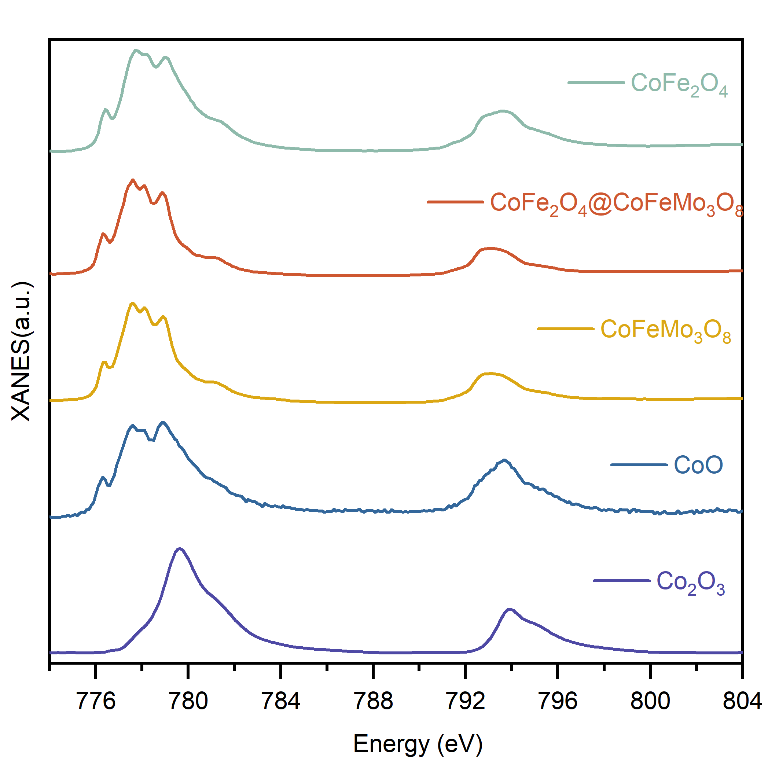


**Figure S7** Co L-edge XANES spectra for as-prepared samples and standard references.


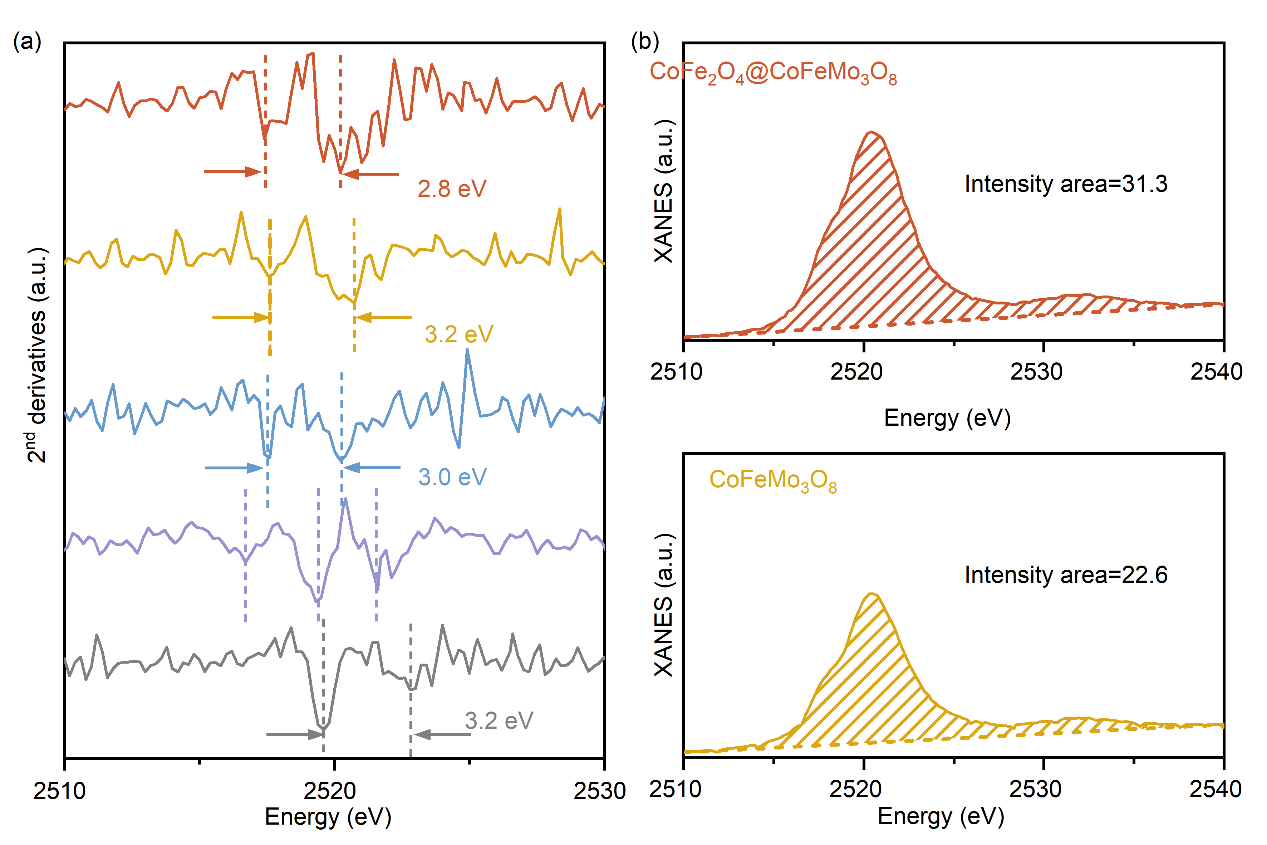


**Figure S8** (a) Second-derivative curves of the Mo L_3_-edge for as-prepared samples and standard references; (b) Demonstration of peak area of Mo L_3_-edge of CoFe_2_O_4_@CoFeMo_3_O_8_ and CoFeMo_3_O_8_.


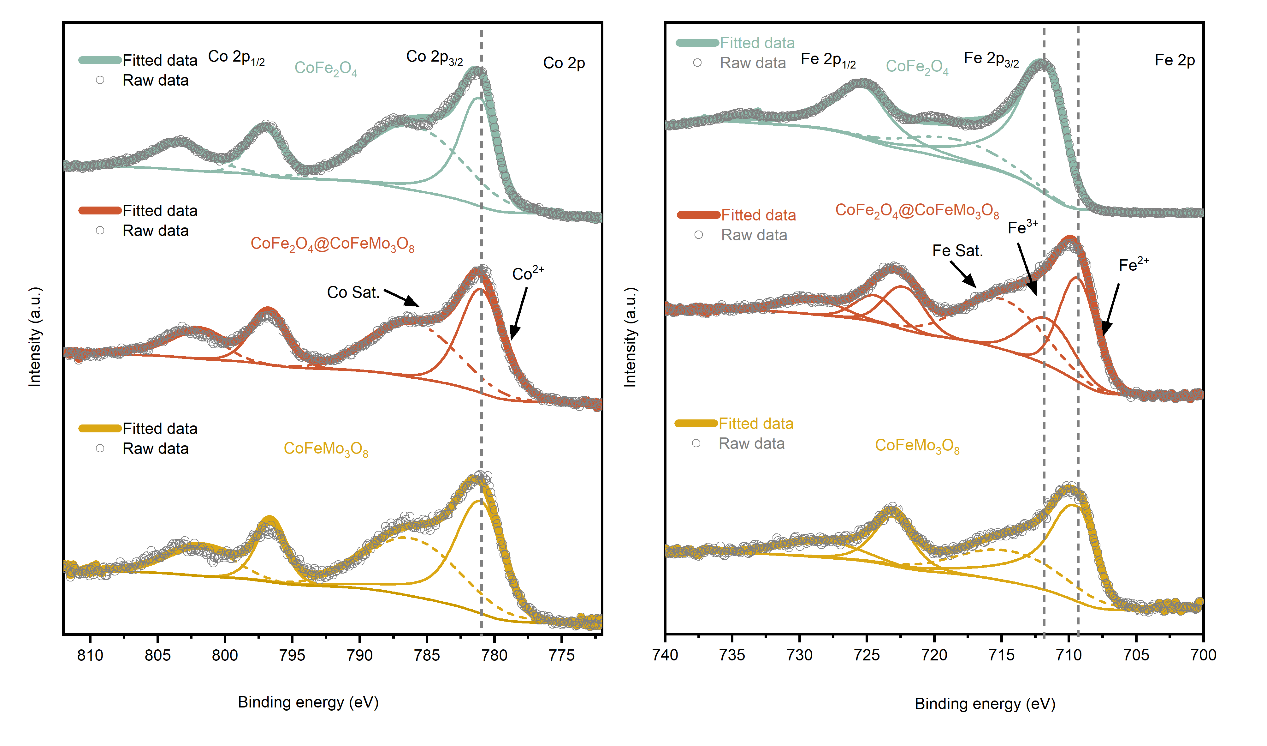


**Figure S9** XPS spectra of (a) Co 2p; (b) Fe 2p of CoFeMo_3_O_8_, CoFe_2_O_4_@CoFeMo_3_O_8_ and CoFe_2_O_4_.


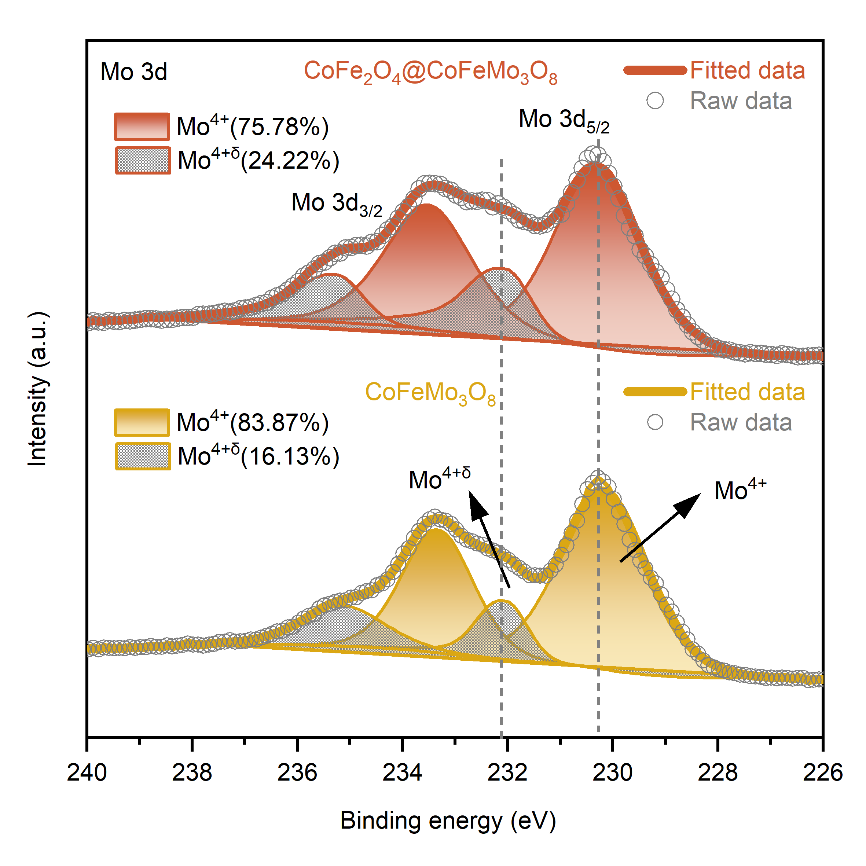


**Figure S10** XPS spectra of Mo 3d of CoFeMo_3_O_8_ and CoFe_2_O_4_@CoFeMo_3_O_8_.


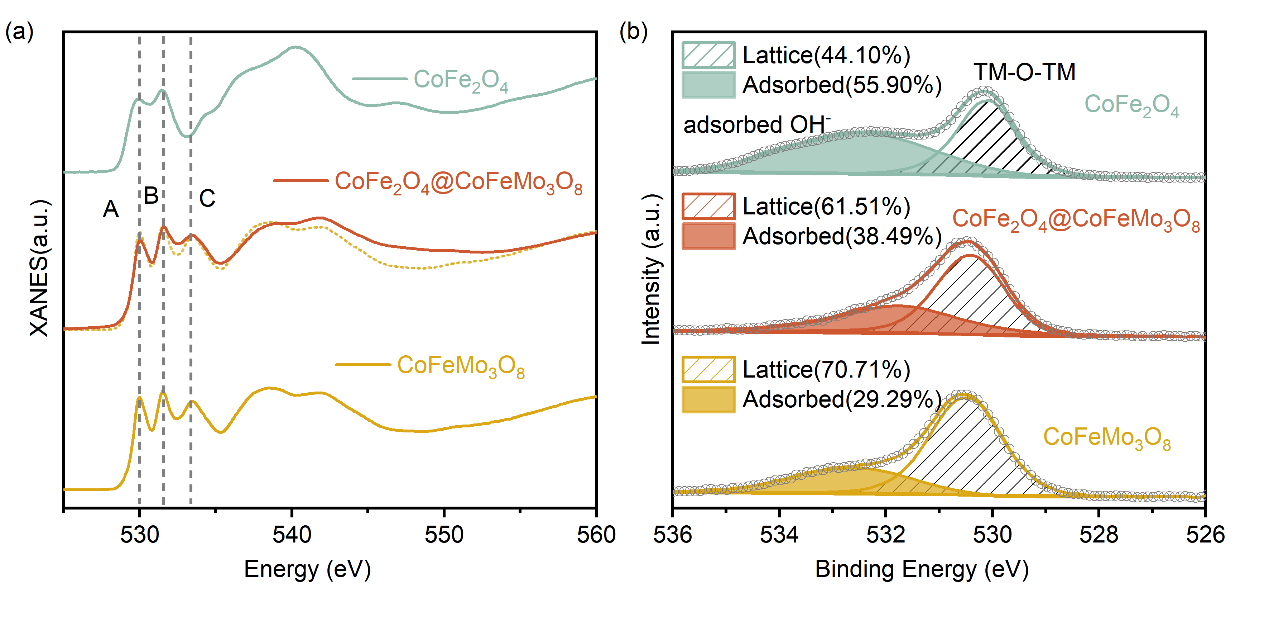


**Figure S11** (a) O K-edge XANES of CoFe_2_O_4_@CoFeMo_3_O_8_ and CoFeMo_3_O_8_; (b) Fitted XPS core level O 1s spectra of CoFe_2_O_4_@CoFeMo_3_O_8_ and CoFeMo_3_O_8_.


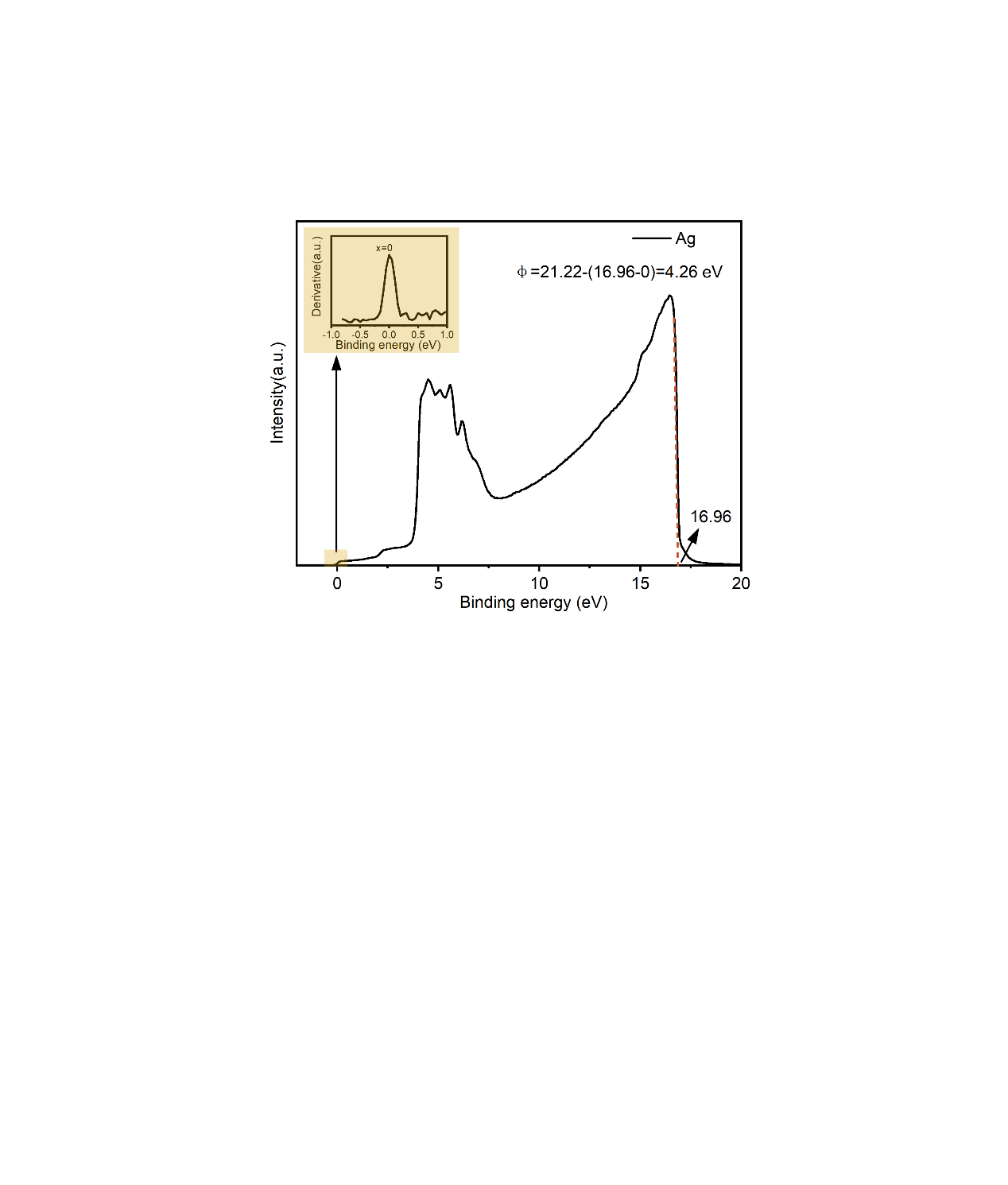


**Figure S12** UPS spectra of Ag for calibration.


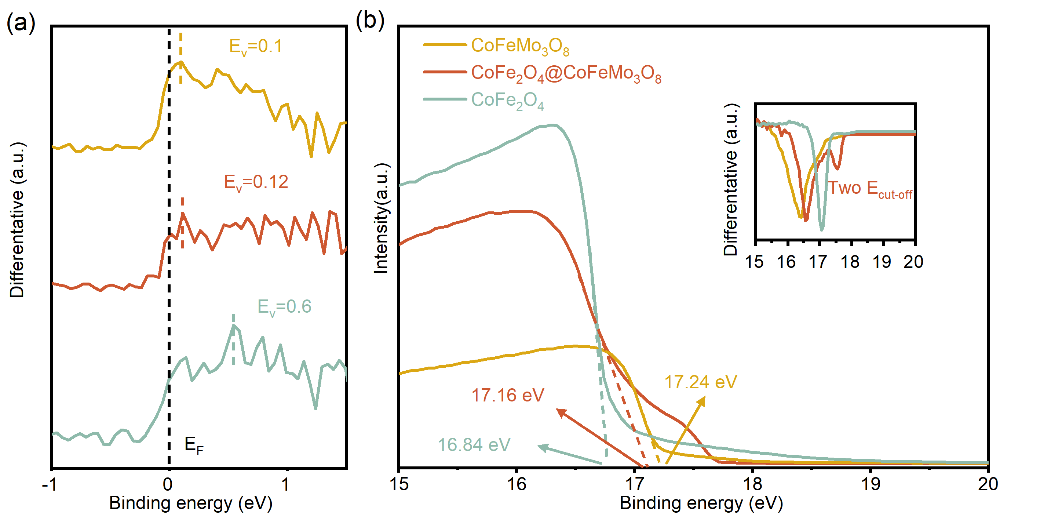


**Figure S13** (a) Differentiated energy distribution curves of CoFeMo_3_O_8_, CoFe_2_O_4_@CoFeMo_3_O_8_ and CoFe_2_O_4_ to estimate the valence band onset of the UPS spectra in the range of low BE; (b) the enlargement of UPS spectra in the high BE range, with the inset showing the corresponding differentiated curve to obtain the cutoff energy.


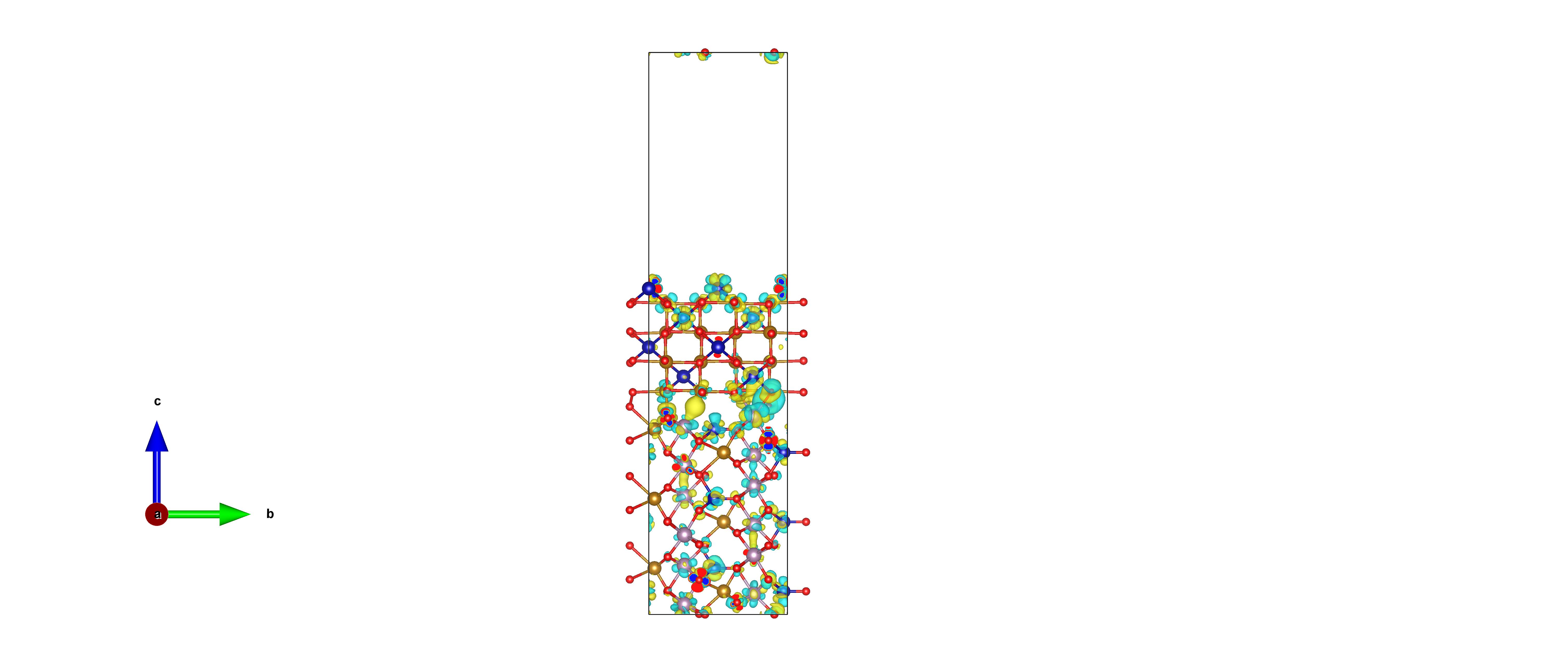


**Figure S14** Charge density difference plot at the CoFe_2_O_4_@CoFeMo_3_O_8_ interface.


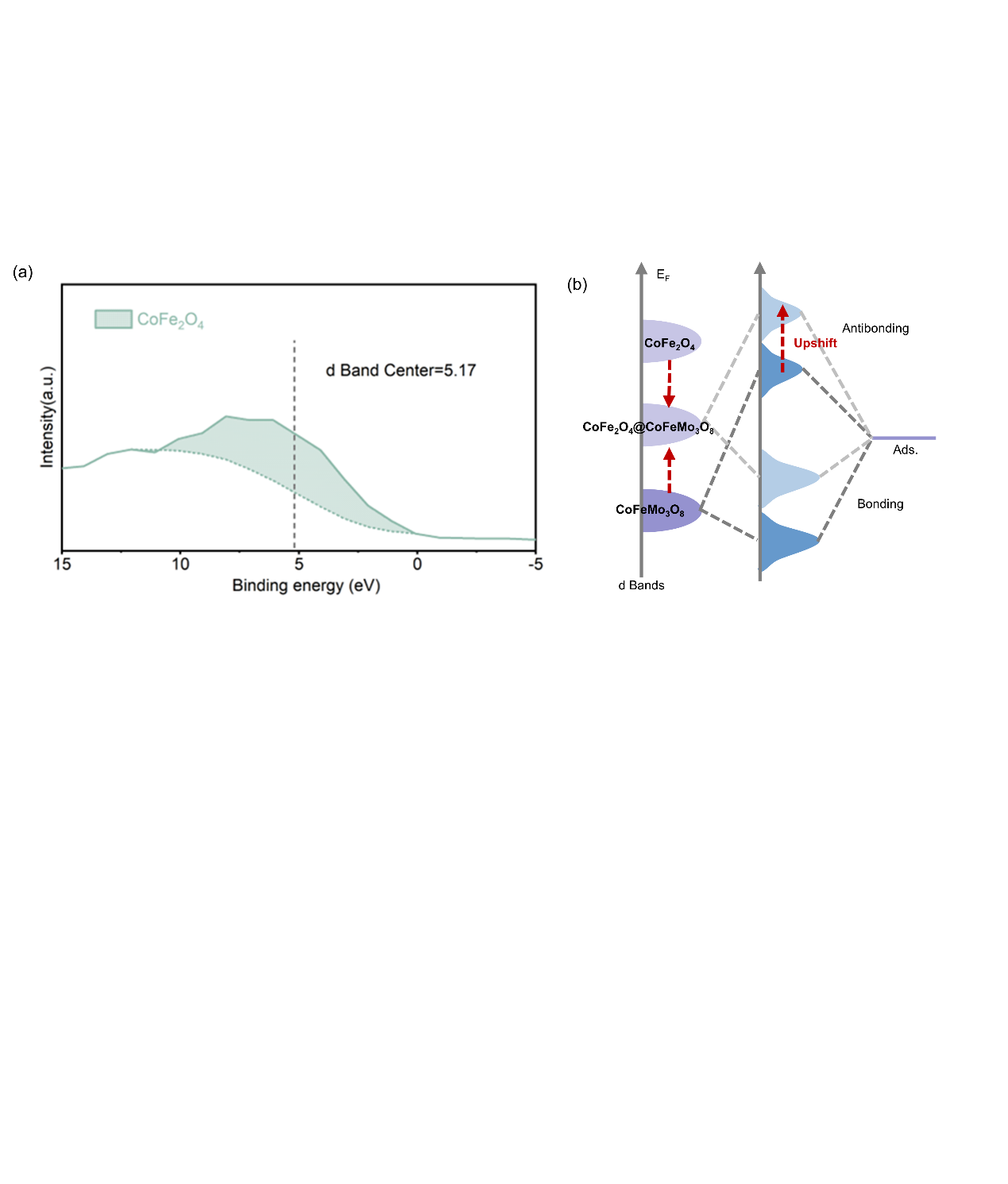


**Figure S15** (a) Valence band spectra of CoFe_2_O_4_; (b) Schematic illustration of the interaction between d bands of catalysts and adsorbed species.

Typically, an upward shift of the d-band center corresponds to an increase in energy within the antibonding orbitals, which in turn results in a reduced electron occupancy in these orbitals. Consequently, the d electrons become more stable with upward d band, leading to the stronger binding energies (Fig. S15(b)). Therefore, the moderate position relative to the Fermi energy of the d-band center in the heterojunction implies neither weak or strong adsorption for reaction intermediates, which is also confirmed by XPS of O 1s in Fig. S11(b).


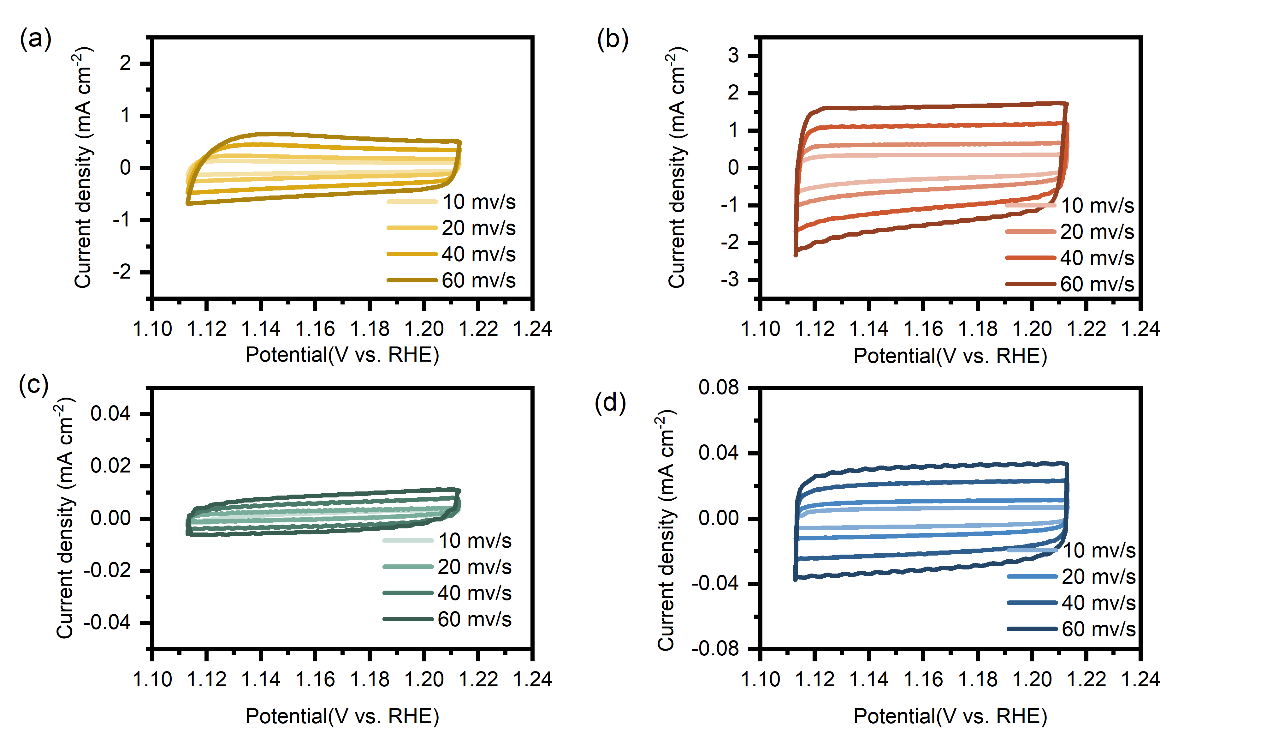


**Figure S16** CV curves of (a) CoFeMo_3_O_8_, (b) CoFe_2_O_4_@CoFeMo_3_O_8_, (c) CoFe_2_O_4_ and (d) IrO_2_, respectively, collected with different scan rates normalized by electrode area.


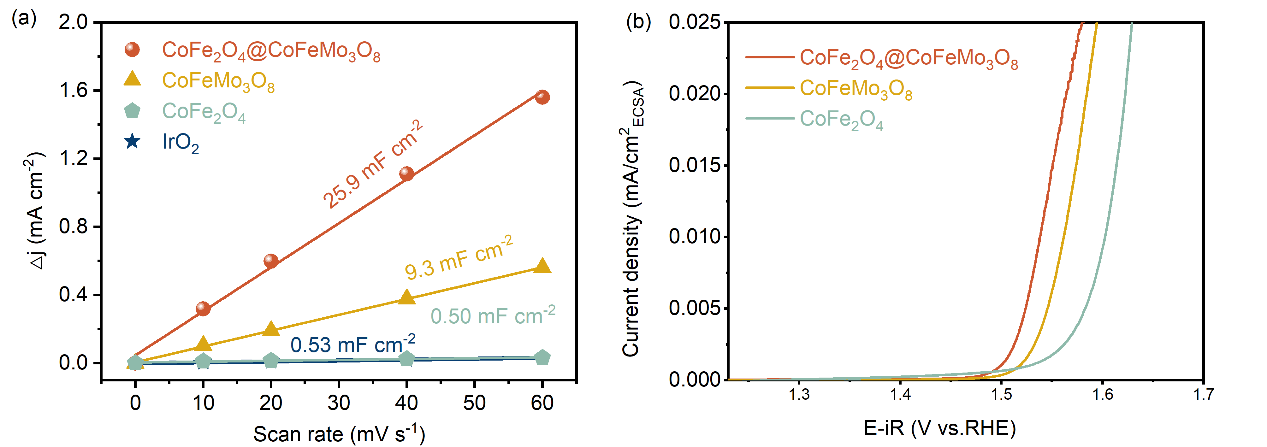


**Figure S17** (a) Current density at 1.16 V at different scan rates for the calculation of double layer capacitance (C_dl_); (b) LSV curves with the current density normalized by ECSA.


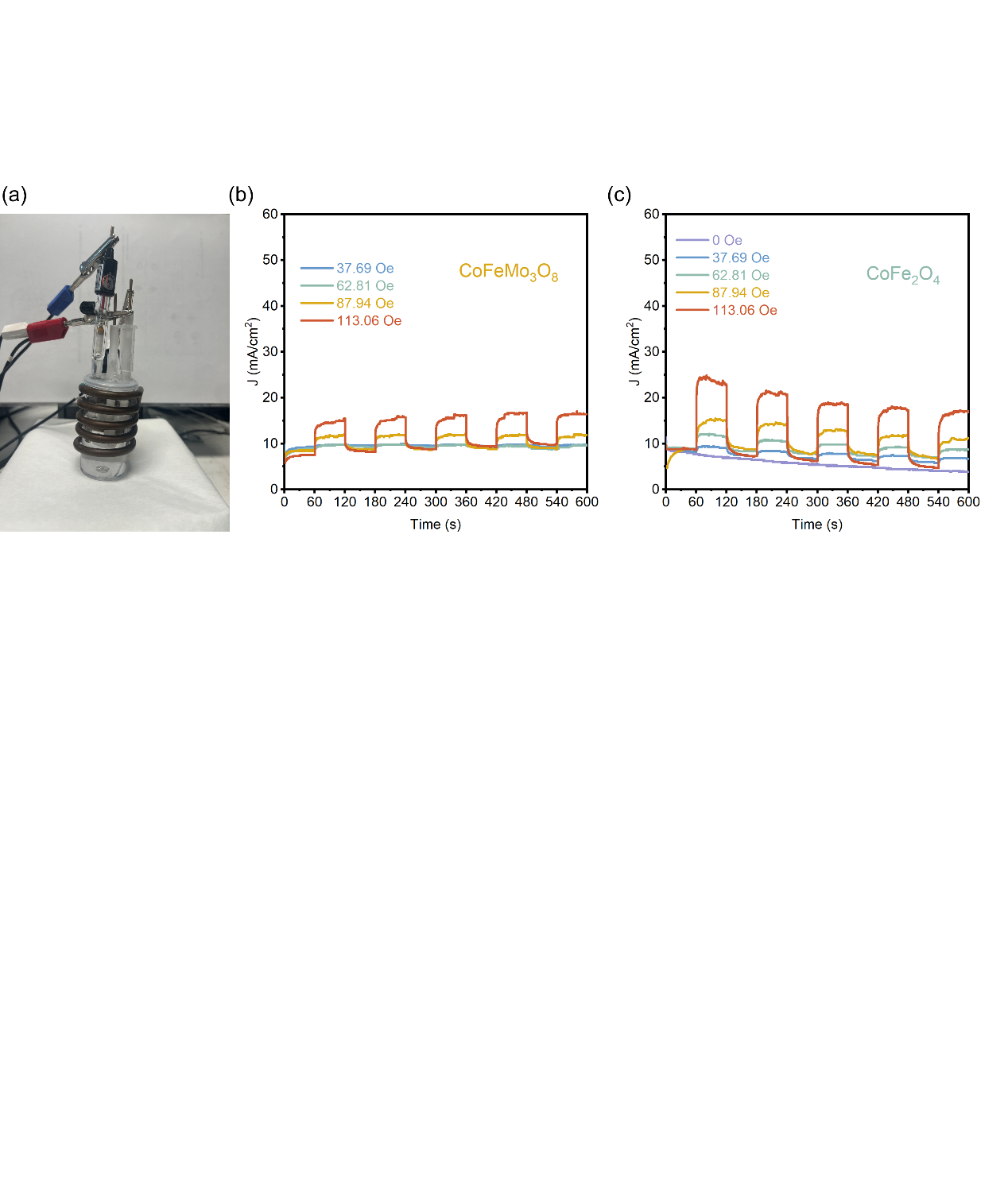


**Figure S18** (a)The diagram of AMF-assisted electrochemical setup; (b) Respond of CoFeMo_3_O_8_ and CoFe_2_O_4_ under AMF treatment in OER.


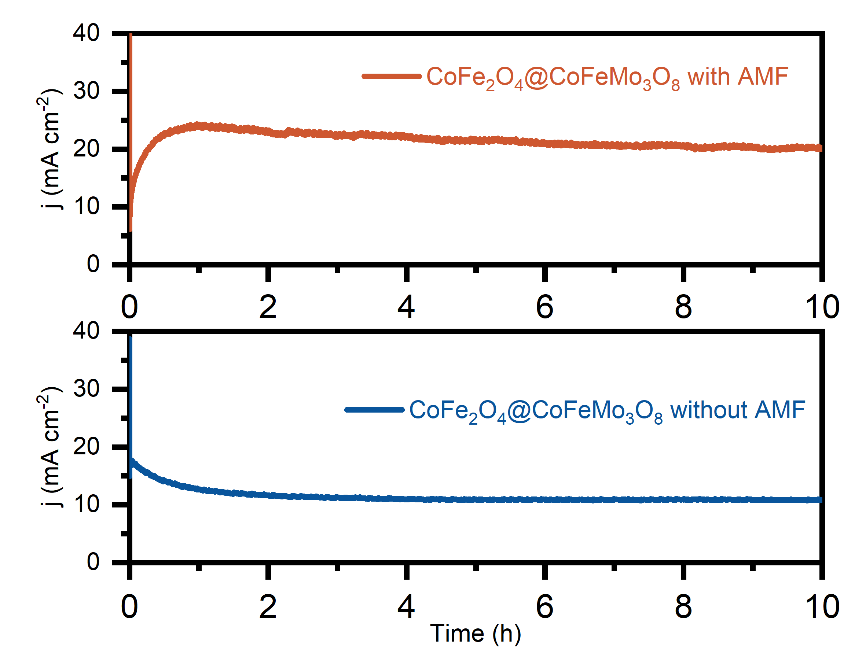


**Figure S19** Long-term chronoamperometric measurement of CoFe_2_O_4_@CoFeMo_3_O_8_ with and without AMF at the potential of 1.50 V versus RHE.


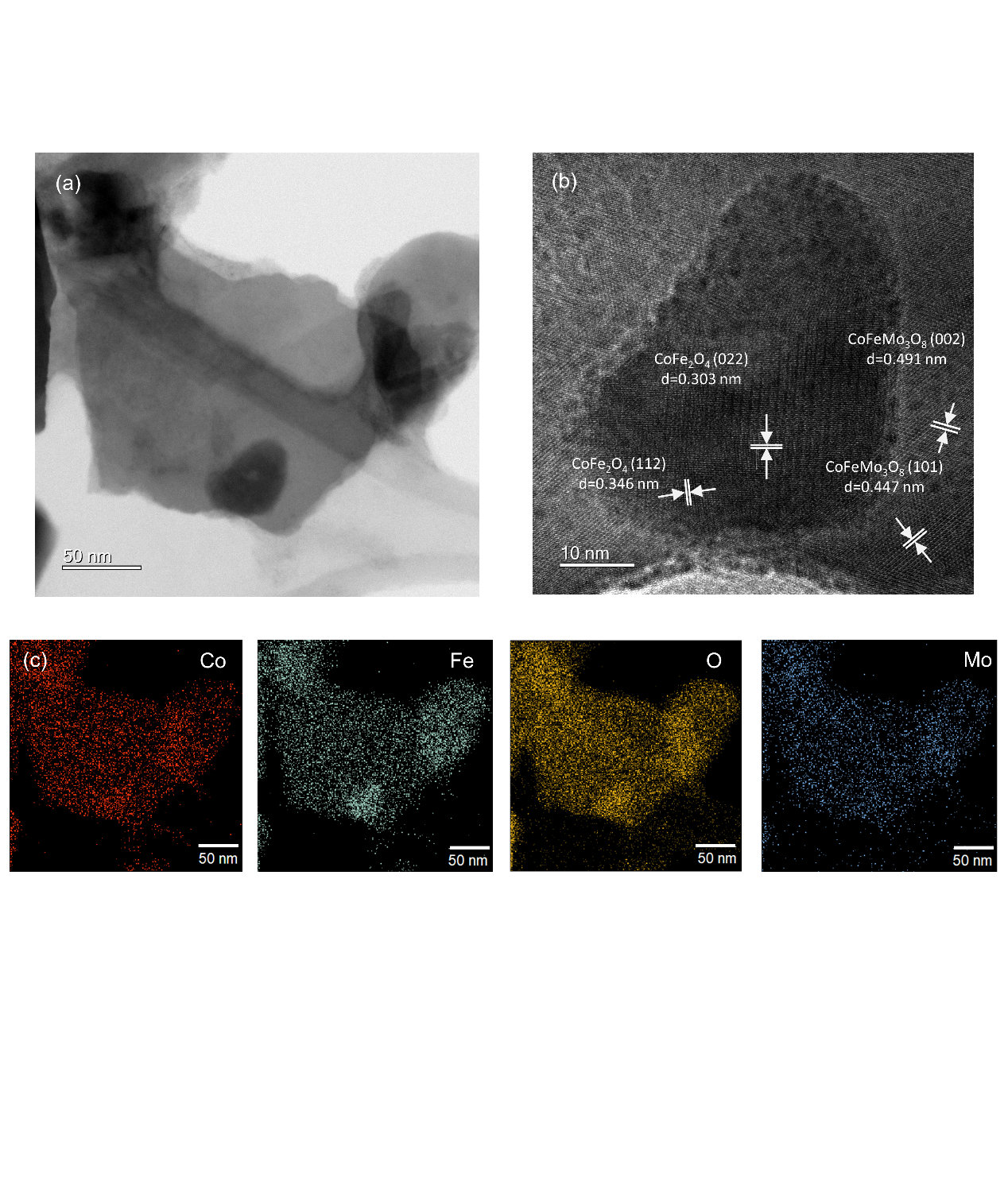


**Figure S20** TEM, HRTEM and EDS mapping images of the CoFe_2_O_4_@CoFeMo_3_O_8_ after OER.


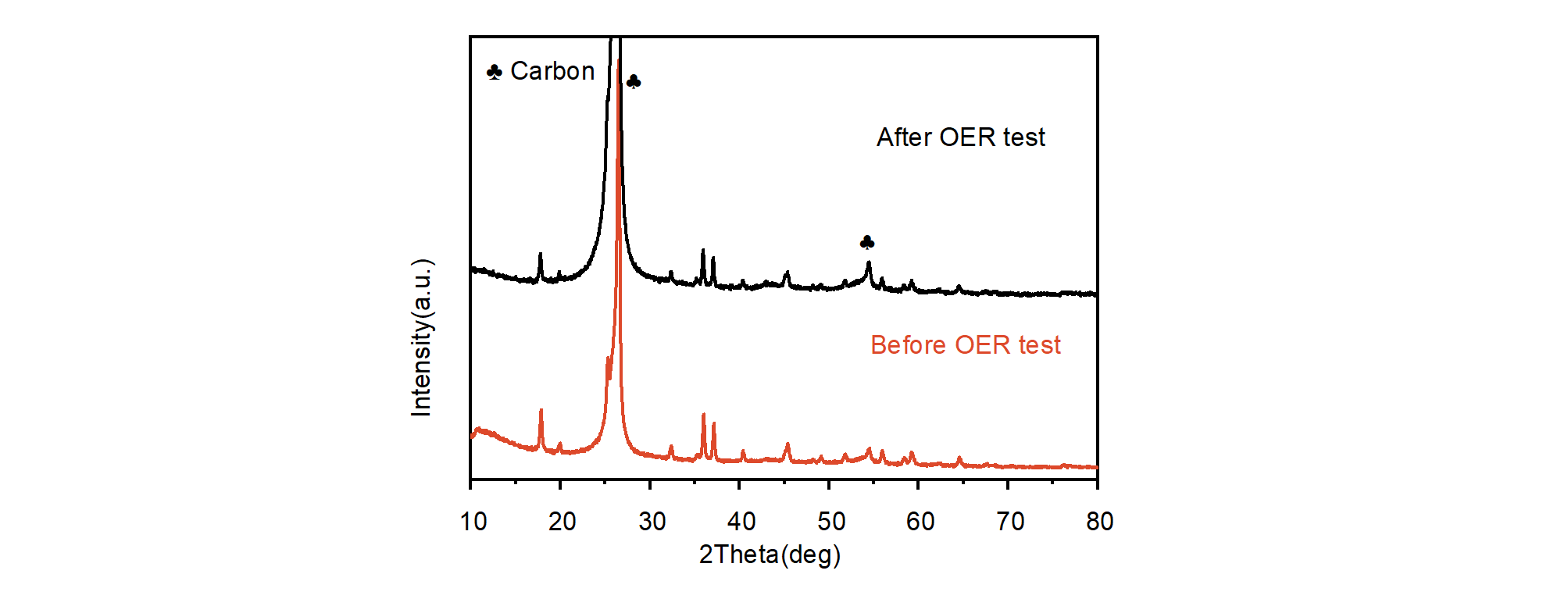


**Figure S21** XRD patterns of the CoFe_2_O_4_@CoFeMo_3_O_8_ attached on the carbon paper electrodes before and after OER tests.


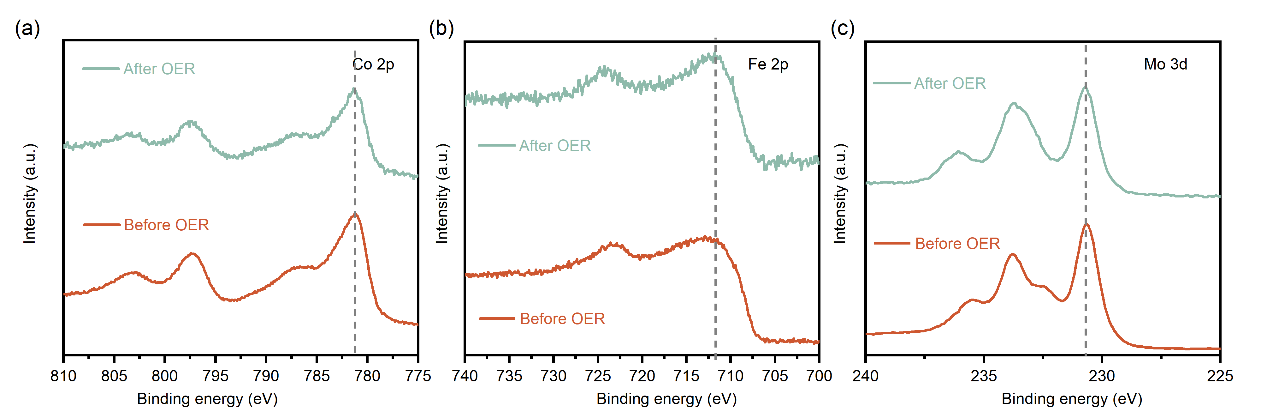


**Figure S22** XPS spectra of (a) Co 2p; (b) Fe 2p; (c) Mo 3d from CoFe_2_O_4_@CoFeMo_3_O_8_ attached on the carbon paper electrodes before and after OER tests.

**Supplementary Tables**

**Table S1** Lattice parameters from the XRD refinement.

| Sample | a=b (Å) | c (Å) | V (Å^3^) |
| --- | --- | --- | --- |
| CoFeMo_3_O_8_ | 5.7908(1) | 9.8878 (9) | 287.15(3) |
| CoFe_2_O_4_@CoFeMo_3_O_8_ | 5.7831(3)  8.4671(0) | 9.9962(9)  8.4671 (0) | 289.53 (1)  607.02(1) |
| CoFe_2_O_4_ | 8.3944(6) | 8.3944(6) | 591.53 (4) |

**Table S2** Fitting parameters of O 1s core-level spectra.

| Sample | Peak1 (eV) | Ratio | Peak2 (eV) | Ratio |
| --- | --- | --- | --- | --- |
| CoFeMo_3_O_8_ | 532.68 | 29.29 | 530.48 | 70.71 |
| CoFe_2_O_4_@CoFeMo_3_O_8_ | 531.78 | 38.49 | 530.38 | 61.51 |
| CoFe_2_O_4_ | 532.50 | 55.90 | 530.06 | 44.10 |

**Table S3** Parameters by analyze Nyquist plots.

| Sample | R_p_ (Ω) | R_s_ (Ω) | T(CPE) (S·s^p^/cm^2^) | P (CPE) |
| --- | --- | --- | --- | --- |
| CoFeMo_3_O_8_ | 27.64 | 9.521 | 9.8833×10^-4^ | 0.70877 |
| CoFe_2_O_4_@CoFeMo_3_O_8_ | 25.13 | 10.09 | 4.9817×10^-3^ | 0.88977 |
| CoFe_2_O_4_ | 65.35 | 10.85 | 1.5886×10^-4^ | 0.59789 |
| Phy mix | 13.26  45.32 | 11.74 | 1.6104×10^-4^  2.207×10^-5^ | 0.81491  0.76601 |
| IrO_2_ | 113.9 | 13.1 | 5.4616×10^-5^ | 0.85305 |

**Table S4** Comparisons of OER performances for CoFe_2_O_4_@CoFeMo_3_O_8_ with previously reported catalysts.

| Electrocatalysts | Tafel slop (mV dec^-1^) | | η(mV) | Loading amount | Refs. |
| --- | --- | --- | --- | --- | --- |
| CoFe_2_O_4_@CoFeMo_3_O_8_  without AMF | 35.14 | | η_10_=270 | 0.386 mg cm^-2^ | This work |
| CoFe_2_O_4_@CoFeMo_3_O_8_  with AMF | 26.06 | | η_10_=240 | 0.386 mg cm^-2^ | This work |
| FeCoMo_3_O_8_ | 43.7 | | η_10_=308 | 0.386 mg cm^-2^ | ^[9]^ |
| Co_2_Mo_3_O_8_@NC-800 | 87.5 | | η_10_=331 | 0.14 mg cm^-2^ | ^[10]^ |
| Co/Co_2_Mo_3_O_8_@NC | | 86.4 | η_10_=288 | 0.26 mg cm^-2^ | ^[11]^ |
| Fe-doped Co_2_Mo_3_O_8_/MoO_3_/Co_3_O_4_/NF | | 52.1 | η_10_=289 | 1 mg cm^-2^ | ^[12]^ |
| Co_2_Mo_3_O_8_/Nb_2_O_5_ | | 88.75 | η_10_=284 | 1.6 mg cm^-2^ | ^[13]^ |
| MoO_2_–Co_2_Mo_3_O_8_@C | | 88 | η_10_=320 | 0.2 mg cm^-2^ | ^[14]^ |
| Fe-doped MoS_2_/CoMo_2_S_4_ | | 65 | η_10_=290 | 0.283 mg cm^-2^ | ^[15]^ |
| Co_3_O_4_(111) | | 27 | η_10_=330 | 0.38 mg cm^-2^ | ^[16]^ |
| Co_3_O_4_-V_Co_ | | 38.2 | η_10_=262 | 0.34 mg cm^-2^ | ^[17]^ |
| CoFe_2_O_4_/Co(Fe)O_x_H_y_ | | 44 | η_10_=300 | 0.255 mg cm^−2^ | ^[18]^ |
| CoFe_2_O_4_ | | 87.8 | η_10_=390 | 0.500 mg cm ^−2^ | ^[19]^ |

**Supplementary Note1 |** **X-ray Absorption spectroscopy analysis**

**Mo L_3_ edge**

In general, for Mo L_3_-edge, the white line edge is split into a doublet due to ligand field splitting. The second derivatives of the edge are therefore quite informative, resolving the doublet splitting values as shown in Fig. S9(a). Generally, the magnitude of the splitting in tetrahedral symmetry (△e,t_2_ split ≤ 2.4 eV) is far less that in an octahedral field (△t_2g_,e_g_ split ≥ 3.0 eV)^[20–22]^. It is obvious that the splitting of the peaks (3.2 eV and 2.8 eV) indicates that the Mo centers within CoFeMo_3_O_8_ and CoFe_2_O_4_@CoFeMo_3_O_8_ are both octahedrally coordinated. The number of available orbitals for t_2g_ and e_g_ are also reflected in the relative intensity of these white lines^[23]^. The branching ratios of t_2g_ and e_g_ in CoFe_2_O_4_@CoFeMo_3_O_8_ are slightly higher than that seen in CoFeMo_3_O_8_, implying more unoccupied t_2g_ states in the heterostructure CoFe_2_O_4_@CoFeMo_3_O_8_. Additionally, the integrated intensity of white line reflects the number of the unoccupied d orbitals^[24]^. The larger intensity (area) of the white line area observed in CoFe_2_O_4_@CoFeMo_3_O_8_ compared to CoFeMo_3_O_8_ as shown in Fig. S9(b), suggests that the presence of CoFe_2_O_4_ can increase the density of unoccupied states in Mo atoms.

**O K edge**

The orbital hybridization between O 2p and the transition metal orbitals 3d/4d is represented in the O K-edge XANES spectra in Fig. S11(a). The first two peaks A and B at around 530.0 eV and 531.5 eV, which are split by the crystal field, can be attributed to the excitation of O 1s states to hybridized O 2p-Co/Fe 3d states, whilst peak C (533.5 eV) corresponds to oxygen 2p hybridized with Mo 4d states^[9]^. The high energy features (centered at about 539 eV and 542 eV) are dominated by Co and/or Fe 4sp states and Mo 5sp states with a wider bandwidth^[25]^. For enhanced visibility, the O K edge of CoFeMo_3_O_8_ is delineated with a yellow dashed line in the context of the CoFe_2_O_4_@CoFeMo_3_O_8_ heterojunction, as illustrated in Fig. S11(a). The spectral intensity, which serves as an indicator of the number of empty states in the respective orbitals^[25]^, shows a slight reduction in the ratio of A to B (unoccupied low-energy orbitals to unoccupied high-energy orbitals) for CoFe_2_O_4_@CoFeMo_3_O_8_. This decrease suggests the emergence of a minuscule number of lower spin states of Co and/or Fe upon the formation of the heterojunction. The hybridization of O and the TM can be further estimated by comparing the pre-peak areas in the O K-edge spectra. This estimation is based on the assumption that the spectral intensity is linearly proportional to the total number of unoccupied states^[26]^. Consequently, the enhanced peak area seen in CoFe_2_O_4_@CoFeMo_3_O_8_ suggests that stronger hybridization of unoccupied O 2p-TM 3d states occurs for the heterostructure than for CoFeMo_3_O_8_. This enhancement is conducive to improved oxygen electrocatalysis, which is driven by the need to extract electrons from oxygen during electrocatalysis^[27]^.

**Supplementary Note2 |** **Ultraviolet based spectroscopy analysis**

**Ultraviolet Photoemission Spectroscopy (UPS)**

The valence band maximum (VBM) position relative to the Fermi level (E_F_) is determined by the binding energy (BE) at which photoelectron signal emerge, whilst the material’s work function (WF) is obtained by subtracting the cutoff energy of escaped secondary electron (E_cut-off_ ) from the incident photon energy (hv =21.22 eV) ^[26,28]^. After establishment of the Fermi energy position using a standard Ag substrate as shown in Fig. S12, the VBM relative to E_F_ of CoFeMo_3_O_8_, CoFe_2_O_4_@CoFeMo_3_O_8_ and CoFe_2_O_4_ are revealed as 0.10 eV, 0.12 eV and 0.60 eV, respectively (Fig. S13(a)). The purple shading at the high binding energy range in Fig. 2(d) represents the secondary electron cut-off region. Based on the following Equation: WF= (hν − E_cut−off_), the WF is estimated to be 3.98 eV, 4.08 eV, and 4.38 eV for the as-prepared CoFeMo_3_O_8_, CoFe_2_O_4_@CoFeMo_3_O_8_ and CoFe_2_O_4_, respectively (Fig. S13(b)). Remarkably, two cutoff edges are observed in the CoFe_2_O_4_@CoFeMo_3_O_8_ with two minimum points in the derivative curves in the inset of Fig. S13(b), which is attributed to the electric field induced by surface dipoles^[28,29]^.

**Ultraviolet-visible spectroscopy (UV-Vis)**

According to Tauc’s relation: (αhν)^1/2^ = B(hν - E_g_), where h represents the Planck constant, ν is the photon frequency, E_g_ is the band gap energy, and α and B are related constants, the value of the indirect optical energy gap can be determined by extrapolating the line segment of the plot to intersect the hν-axis.

References

[1] C. C. L. McCrory, S. Jung, J. C. Peters, T. F. Jaramillo, *J. Am. Chem. Soc.* **2013**, *135*, 16977.

[2] X. Sun, Q. Shao, Y. Pi, J. Guo, X. Huang, *J. Mater. Chem. A* **2017**, *5*, 7769.

[3] A. T. Swesi, J. Masud, M. Nath, *Energy Environ. Sci.* **2016**, *9*, 1771.

[4] G. Kresse, J. Furthmüller, *Comp. mater. sci.* **1996**, *6*, 15.

[5] L. Chaput, P. Pécheur, H. Scherrer, *Phys. Rev. B* **2007**, *75*, 045116.

[6] J. Tao, J. P. Perdew, H. Tang, C. Shahi, *J. Chem. Phys.* **2018**, *148*, 074110.

[7] S. L. Dudarev, G. A. Botton, S. Y. Savrasov, C. J. Humphreys, A. P. Sutton, *Phys. Rev. B* **1998**, *57*, 1505.

[8] W. Tang, E. Sanville, G. Henkelman, *J. Phys.: Condens. Matter* **2009**, *21*, 084204.

[9] C. Hao, X. Li, H. Huang, L. Ge, Z. Fu, Y. Lu, Y. Wang, S. Zhang, Z. Cheng, *ACS Energy Lett.* **2023**, *8*, 4506.

[10] T. Ouyang, X. Wang, X. Mai, A. Chen, Z. Tang, Z. Liu, *Angew. Chem. Int. Ed.* **2020**, *59*, 11948.

[11] Y. Zhang, W. Ye, J. Fan, V. Cecen, P. Shi, Y. Min, Q. Xu, *ACS Sustain. Chem. Eng.* **2021**, *9*, 11052.

[12] W. Liu, W. Que, R. Yin, J. Dai, D. Zheng, J. Feng, X. Xu, F. Wu, W. Shi, X. Liu, X. Cao, *Appl. Catal. B: Environ.* **2023**, *328*, 122488.

[13] L. Ye, Z.-J. Jiang, Z. Jiang, *J. Alloys Compd.* **2023,** *963*, 171266.

[14] Y. Li, H. Xu, H. Huang, C. Wang, L. Gao, T. Ma, *Chem. Commun.* **2018**, *54*, 2739.

[15] Y. Guo, J. Tang, J. Henzie, B. Jiang, W. Xia, T. Chen, Y. Bando, Y.-M. Kang, Md. S. A. Hossain, Y. Sugahara, Y. Yamauchi, *ACS Nano* **2020**, *14*, 4141.

[16] X. Li, L. Ge, Y. Du, H. Huang, Y. Ha, Z. Fu, Y. Lu, W. Yang, X. Wang, Z. Cheng, *ACS Nano* **2023**, *17*, 6811-6821.

[17] R. Zhang, L. Pan, B. Guo, Z.-F. Huang, Z. Chen, L. Wang, X. Zhang, Z. Guo, W. Xu, K. P. Loh, J.-J. Zou, *J. Am. Chem. Soc.* **2023**, *145*, 2271.

[18] T. Wu, X. Ren, Y. Sun, S. Sun, G. Xian, G. G. Scherer, A. C. Fisher, D. Mandler, J. W. Ager, A. Grimaud, J. Wang, C. Shen, H. Yang, J. Gracia, H.-J. Gao, Z. J. Xu, *Nat. Commun.* **2021**, *12*, 3634.

[19] X. Ren, T. Wu, Y. Sun, Y. Li, G. Xian, X. Liu, C. Shen, J. Gracia, H.-J. Gao, H. Yang, Z. J. Xu, *Nat. Commun.* **2021**, *12*, 2608.

[20] S. R. Bare, G. E. Mitchell, J. J. Maj, G. E. Vrieland, J. L. Gland, *J. Phys. Chem.* **1993**, *97*, 6048.

[21] J. Evans, J. F. W. Mosselmans, *J. Phys. Chem.* **1991**, *95*, 9673.

[22] H. Aritani, S. Shinohara, S. Koyama, K. Otsuki, T. Kubo, A. Nakahira, *Chem. Lett.* **2006**, *35*, 416.

[23] H. Hu, I. E. Wachs, S. R. Bare, *J. Phys. Chem.* **1995**, *99*, 10897.

[24] E. J. Lede, F. G. Requejo, B. Pawelec, J. L. G. Fierro, *J. Phys. Chem. B* **2002**, *106*, 7824.

[25] F. Frati, M. O. J. Y. Hunault, F. M. F. de Groot, *Chem. Rev.* **2020**, *120*, 4056.

[26] X. Li, Y. Bai, Z. Cheng, *Adv. Sci.* **2021**, *8*, 2101000.

[27] J. Suntivich, W. T. Hong, Y.-L. Lee, J. M. Rondinelli, W. Yang, J. B. Goodenough, B. Dabrowski, J. W. Freeland, Y. Shao-Horn, *J. Phys. Chem. C* **2014**, *118*, 1856.

[28] X. Li, Y. Du, L. Ge, C. Hao, Y. Bai, Z. Fu, Y. Lu, Z. Cheng, *Adv. Funct. Mater.* **2022**, 2210194.

[29] T. Schultz, T. Lenz, N. Kotadiya, G. Heimel, G. Glasser, R. Berger, P. W. M. Blom, P. Amsalem, D. M. de Leeuw, N. Koch, *Ad. Mater. Interfaces* **2017**, *4*, 1700324.
